# A Meta-Analysis and Systematic Review of *Listeria monocytogenes* Response to Sanitizer Treatments

**DOI:** 10.3390/foods12010154

**Published:** 2022-12-28

**Authors:** Minmin Hu, Qingli Dong, Yangtai Liu, Tianmei Sun, Mingliang Gu, Huajian Zhu, Xuejuan Xia, Zhuosi Li, Xiang Wang, Yue Ma, Shuo Yang, Xiaojie Qin

**Affiliations:** 1 School of Health Science and Engineering, University of Shanghai for Science and Technology, Shanghai 200093, China; 2 College of Environmental Science and Engineering, Ocean University of China, Qingdao 266100, China

**Keywords:** foodborne pathogen, *Listeria monocytogenes*, sanitizer, resistance, inactivation, the mixed-effects model

## Abstract

*Listeria monocytogenes* is a ubiquitous organism that can be found in food-related environments, and sanitizers commonly prevent and control it. The aim of this study is to perform a meta-analysis of *L. monocytogenes* response to sanitizer treatments. According to the principle of systematic review, we extracted 896 records on the mean log-reduction of *L. monocytogenes* from 84 publications as the dataset for this study. We applied a mixed-effects model to describe *L. monocytogenes* response to sanitizer treatment by considering sanitizer type, matrix type, biofilm status, sanitizer concentration, treatment time, and temperature. Based on the established model, we compared the response of *L. monocytogenes* under different hypothetical conditions using forest plots. The results showed that environmental factors (i.e., sanitizer concentration, temperature, and treatment time) affected the average log-reduction of *L. monocytogenes* (*p* < 0.05). *L. monocytogenes* generally exhibited strong resistance to citric acid and sodium hypochlorite but had low resistance to electrolyzed water. The planktonic cells of *L. monocytogenes* were less resistant to peracetic acid and sodium hypochlorite than the adherent and biofilm cells. Additionally, the physical and chemical properties of the contaminated or inoculated matrix or surface also influenced the sanitizer effectiveness. This review may contribute to increasing our knowledge of *L. monocytogenes* resistance to sanitizers and raising awareness of appropriate safety precautions.

## 1. Introduction

*Listeria monocytogenes*, a Gram-positive bacterium, is widely distributed and persists in contaminated foods and food-related environments [1]. It is also a typical foodborne pathogen that can cause human invasive listeriosis, leading to meningitis, abortion, or even death [2,3]. Many reports on food safety regarding *L. monocytogenes* exist [4,5]. From 1 January 2017, to 17 July 2018, a total of 1060 cases were reported in South Africa. According to the World Health Organization (WHO), this was the biggest listeriosis outbreak that ever occurred in the world [6]. Thus, applying disinfecting and cleaning practices to reduce the possibility of *L. monocytogenes* exposure in the food chain is essential.

Researchers have widely studied sanitizers, such as sodium hypochlorite [7], chlorine dioxide water [8], peroxyacetic acid [9], electrolyzed oxidizing water [10], hydrogen peroxide [11], and surfactant detergent [12], and have demonstrated that these sanitizers are highly efficient at reducing and inactivating *L. monocytogenes* and other pathogens in foods and environments. However, the physiological and ecological behaviors of *L. monocytogenes* perform differently under adverse conditions, including when sanitizers and other bactericidal agents are present [13,14,15,16,17]. The lack of understanding of *L. monocytogenes* response to sanitizers may mislead subsequent control measures.

A meta-analysis is a useful statistical tool to systematically analyze a large collection of data from multiple individual studies. The main characteristic of meta-analyses is the use of statistical methods to quantitatively integrate the results of studies. Currently, meta-analysis is increasingly applied in the field of food microbiology, providing quantitative knowledge based on a cross-sectional survey of microbiological characteristics [18,19,20]. Many authors used meta-analysis to evaluate the interventions in inactivating three foodborne pathogens in fresh produce [21], electrolyzed water treatments in reducing foodborne pathogens on different foods [22], and virus inactivation on hard surfaces or in suspension by chemical disinfectants [23]. This method can be further utilized to comprehensively determine the link between internal/external factors and microbiological responses in various cleaning and disinfection scenarios.

Therefore, the aim of this study was to conduct a meta-analysis to compare and predict the effectiveness of various sanitizers and cleansers on *L. monocytogenes*. The response of *L. monocytogenes* to food-related sanitizers in different situations would be explained by constructing a mixed-effects model. Additionally, with the constructed model, the overall effectiveness on the reduction of *L. monocytogenes* was predicted and compared under different hypothetical conditions.

## 2. Materials and Methods

### 2.1. Search Strategy

To determine our research question, the PICO (population, intervention, control/comparison, and outcome) approach used in the evaluation of evidence-based clinical questions was employed [24]. The population was specified as the system of solutions, foods, and food contact surfaces, as well as the bacterial status of plankton, adhesion, and biofilm. The intervention was conducting by the disinfecting and cleaning treatments on the population. The comparison was the used sanitizers and cleansers, and the outcome was the log reductions of *L. monocytogenes* after disinfecting and cleaning treatments.

The full search query that we used for our literature search in the Web of Science Core Collection was as follows: TS = (detergent OR cleaner OR cleaning agent OR disinfectant OR sanitizer) AND TS = (resistance OR tolerance OR survival OR growth OR inactivation) AND TS = *listeria*. Additionally, the published dates were from 1 January 1985 to 31 December 2021. We found a total of 1200 results (on 11 April 2022).

### 2.2. Selection Criteria and Data Extraction

We performed a preliminary screening based on the title and abstract. We excluded studies with partial topics, such as literature reviews and studies with research objects other than *L. monocytogenes* (such as *L. innocua* or *L. grayi*). We excluded studies wherein the authors used disinfection methods such as nanostructures, ultraviolet lamps, electron beams, and antibacterial coating. We also excluded studies wherein the authors used biological extracts (such as essential oils). We excluded studies wherein the authors examined *Leuconostoc*, *Enterococcus*, *Lactobacillus*, and bacteriophages. In the full-text screening step, we excluded studies wherein the authors used a combination of multiple disinfection or cleaning methods, unclear sanitizer substances, or *L. monocytogenes* subjected to pressure or stress adaptation. We selected studies that were performed in a colony unit log CFU and studies that contained concentration, time, and temperature data. To stabilize the parameter estimation of the regression models, we reduced the sanitizer concentration range via a log transformation.

**Figure 1:**
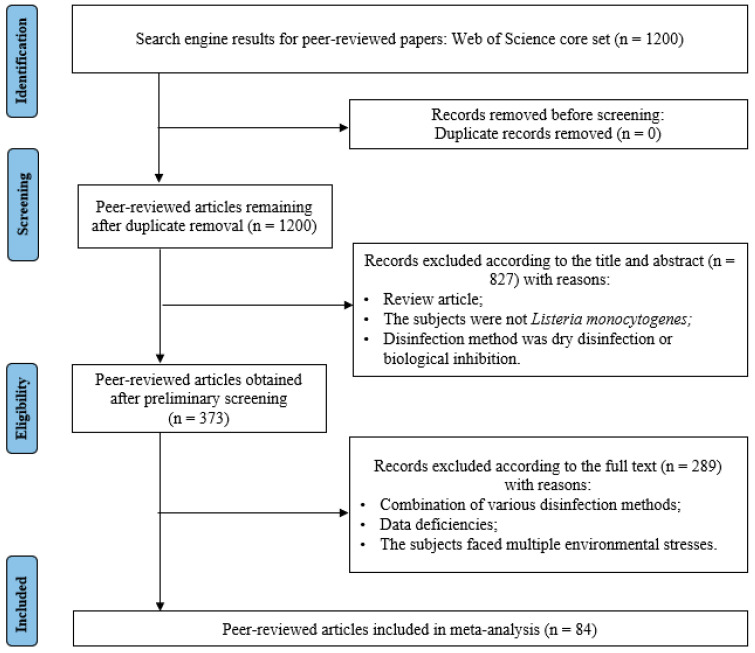
Flow diagram of the systematic review process and the selection of studies for inclusion in the meta-analysis.

The screening rules and process are shown in Figure 1. A total of 84 papers were chosen for the meta-analysis after the final screening. Then, we assembled the experimental settings and observed data in Microsoft Excel (Microsoft, Redmond, WA, USA) using information from the selected research.

### 2.3. Estimation of Summary Effects

Because most studies use the log CFU scale and because it is an intuitively comprehensible parameter, we selected the mean log reduction of *L. monocytogenes* that attained the following treatment with chemical sanitizers as the total effect. Because some studies did not directly provide the log reduction values, we used the following Equation (1) for conversion:

*R = N_b_ − N_a_* (1)

*R* (log CFU/ sample) refers to the reduction of bacteria after the sanitizer treatment; *N_b_* ((log CFU/ sample) refers to the number of bacteria before the sanitizer treatment, and *N_a_* ((log CFU/ sample) refers to the number of bacteria after the sanitizer treatment.

A high level of heterogeneity was anticipated given the variations in testing characteristics, matrices, and analytical techniques found in the studies used in this report. A mixed-effects model is used when estimating the summary effect sizes to account for the high expected heterogeneity levels [25,26,27]. The constructed model is shown in Equation (2):

*R = β_0_ + β_1_Con + β_2_Time + β_3_Temp + ϑ_mn_ + ε_lmn_* (2)

where *β_0_* is the intercept of the fixed effect, *β_1_* is the mean effect of the increment in the 10-base logarithm of the sanitizer concentration (Con, %), *β_2_* is the mean effect of a unit increment in time (Time, min) of the sanitizer, and *β_3_* is the mean effect of a unit increment in the temperature (Temp, °C) of the sanitizer. The parameters *β_0_*, *β_1_*, *β_2_,* and *β_3_* can be viewed as continuous variables describing the sanitizer’s ability to disinfect. This is because the log-reduction *R* for a sanitizer increases with the intercept *β_0_*. Similar to this, for a given concentration, temperature, and time, a sanitizer with higher slopes *β_1_*, *β_2_,* and *β_3_* will result in a higher log-reduction *R*. Due to the diversity of the data structure, considering the influence of the bacteria status *m* and different studies *n* on the results is necessary, so we combined the two variables into an interaction variable (*mn*). We assumed that this interaction would change depending on the random effects *ϑ_mn_* used in Equation (2). Additionally, we assumed a normal distribution for the errors or residuals *ε_lmn_*, with a mean of zero and variance *s^2^*. When interaction effects were examined according to cell status or food contact, matrix *p* and study *n* were considered [21,22]. The letter *l* refers to a certain sanitizer.

### 2.4. Statistic Analysis

We used the “lme” function in the *nlme* package (version 3.1-158, https://cran.r-project.org/web/packages/nlme/index.html, accessed on 18 June 2022) of R (version 4.2.1, https://cran.r-project.org/, accessed on 18 June 2022) to fit the mixed-effects model, and we assigned weights to each primary study in the meta-analyses, typically using the standard errors of the effect sizes as precision measures. However, obtaining the standard error of the log reduction for each study for the current meta-analysis was impossible. Therefore, we modified the precision to mean that it is equivalent to the N sample size employed in each study. For each of the meta-analysis models, we also calculated goodness of fit measures such as the Akaike information criterion (AIC) and Bayesian information criterion (BIC). In addition, for the regression models, we calculated a measure of between-study heterogeneity, *I^2^* [28,29,30]. To further analyze specific scenarios, we subgrouped the data according to the biofilm status and food contact. We used the *metafor* package (version 3.4-0, https://cran.r-project.org/web/packages/metafor/index.html, accessed on 18 June 2022) of R to create forest plots under hypothetical conditions. We defined the statistical significance as *p* < 0.05.

## 3. Results and Discussion

### 3.1. Characteristics of the Extracted Information

Based on the 84 selected studies, we extracted and formatted 896 data items for further analysis (see Table S1). The dataset was cross-examined by all authors, including the records of sanitizer type, sanitizer concentration, treatment time and temperature, strain types, biofilm status, matrix, bacterial log reduction (mean, standard deviation, and replications), and reference records.

**Figure 2:**
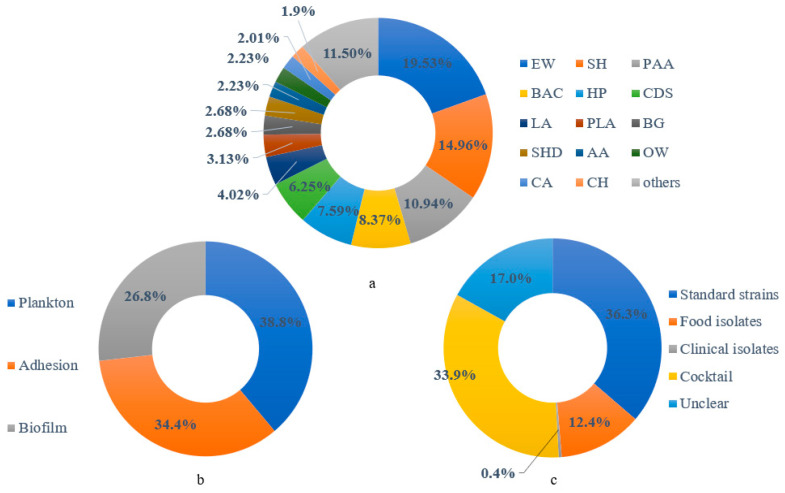
The proportion of (**a**) used sanitizers, (**b**) observed *L. monocytogenes* biofilm statuses, and (**c**) used *L. monocytogenes* strains extracted from the selected studies.

The included papers concern the effect of 36 sanitizers on *L. monocytogenes* response, consisting of electrolyzed water (EW, n = 175), sodium hypochlorite (SH, n = 134), peracetic acid (PAA, n = 98), benzalkonium chloride (BAC, n = 75), hydrogen peroxide (HP, n = 68), chlorine dioxide solution (CDS, n = 56), lactic acid (LA, n = 36), 3-Phenyllactic acid (PLA, n = 28), biguanide (BG, n = 24), sodium hydroxide (SHD, n = 24), ozonated water (OW, n = 20), acetic acid (AA, n = 20), citric acid (CA, n = 18), calcium hypochlorite (CH, n = 17), and others (total n = 103, n < 15/sanitizer) (Figure 2a). The status of *L. monocytogenes* can be classified into planktonic (n = 348), adherent (n = 308), and biofilm (n = 240) (Figure 2b), involving standard strains (n = 325), clinically isolated strains (n = 4), food-isolated strains (n = 111), cocktails (n = 304), and unclear strains (n = 152) (Figure 2c). Most of the studies used standard strains and cocktails (up to 70%), and only 1% of the results were related to the clinically isolated strain. Additionally, they treated all the *L. monocytogenes* with planktonic status in liquid solutions, and they mainly processed the other treatments on solid surfaces, including food and abiotic contact surfaces.

### 3.2. Effect Size Estimation by Sanitizer

We quantitatively measured the tolerance of *L. monocytogenes* under the treatment of different sanitizers using the mixed-effect model of Equation (2). As listed in Table 1, we successfully derived the intercept values and coefficients for six sanitizers. Under the treatment of most sanitizers, the sanitizer concentration, temperature, and treatment time directly affected the log reduction of *L. monocytogenes* (*p* < 0.05). Sanitizers in high concentrations with a high temperature and extended treatment time will help to eliminate *L. monocytogenes* viable cells. We could not estimate the model parameters of some sanitizers very well mainly due to the lack of observations or because the sanitizer was either too weak or too efficient.

Pathogens tend to be less resistant when higher intercepts (*β_0_*) are obtained [21]. The comparison between the estimated intercepts (*β_0_*) indicated that *L. monocytogenes* was more resistant to the CA and SH treatments. In other words, CH, EW, CDS, and PAA may be more effective at inactivating *L. monocytogenes*. These agents (CH, EW, CDS) are chlorine-based sanitizers, which are commonly used to eliminate microorganisms in food-processing environments and water. Chlorine affects bacterial membranes and changes their permeability, causing intracellular materials to change and resulting in fatal DNA damage [31,32]. However, chlorine-based sanitizers may be more unacceptable to consumers when used in food than edible organic acids such as CA. Therefore, current studies are developing new strategies to combine edible acid with other physical treatments to enhance antimicrobial efficiency against foodborne pathogens [33].

### 3.3. Effect Size Estimation by L. monocytogenes Biofilm Status

According to the common status of *L. monocytogenes* in the food chain, we further evaluated the sanitizer effects on planktonic, adherent, and biofilm cells using the mixed-effects models. As the estimated intercepts (*β_0_*) listed in Table 2, we treated the planktonic, adherent, and biofilm cells with a total of six, six, and five different sanitizers, respectively. By comparing the sanitizers’ intercept (*β_0_*) values, we found that the planktonic cells had a low tolerance to the investigated sanitizer (PAA, SH) compared with the adherent and biofilm cells. Similarly, the adherent cells were more resistant than the planktonic cells in the AA sanitizer. From the above description, the planktonic cells were less resistant than the adherent and biofilm cells to most sanitizers, with some exceptions, such as HP. We did not find a consistent sanitizer resistance pattern when we compared the adherent cells with the biofilm cells. The fitted intercepts (*β_0_*) presented in Table 2 also suggest that chlorine-based sanitizers (CDS, EW) appeared to be a kind of effective sanitizer against *L. monocytogenes* regardless of the status. For the biofilm cells, only SH and EW passed the significance test (*p* < 0.05). The original data showed that the HP and LA sanitizer had 14 and 9 data points, respectively, which may lead to the low significance of the intercept (*β_0_*). In other words, the amount of data for biofilm disinfection research was not enough.

**Figure 3:**
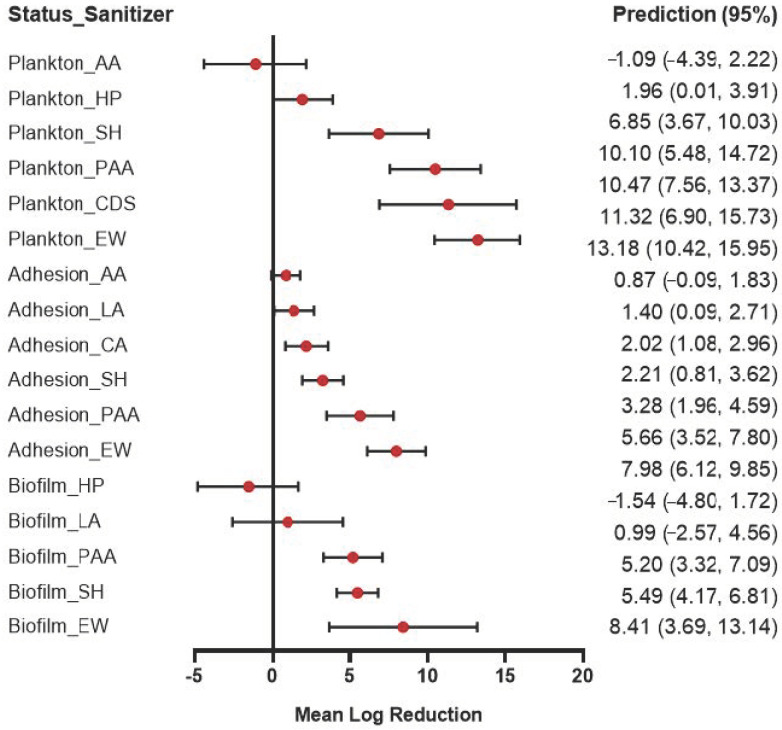
Forest plot of the predicted log reduction of *L. monocytogenes* in plankton, adhesion, and biofilm statuses using a common hypothetical treatment of 1% sanitizers at 15 min, 25 °C.

To horizontally compare the effect of different sanitizers under the same conditions, the fitted meta-analysis models were solved for a hypothetical treatment with 1% sanitizer and exposure time of 15 min at 25 °C. In Figure 3, these predicted values are depicted as forest plots. Under the assumed treatment conditions, we found that AA caused the lowest log reduction in the planktonic cells (−1.09, (−4.39,2.22)) and the adherent cells (0.87, (−0.09,1.83)). In the planktonic cells, the response to different sanitizers fluctuated widely, with the highest being 13.18. The sanitizers (SH, PAA, CDS, EW) at this hypothetical concentration could likely eliminate the planktonic cells. For the biofilm bacteria, we obtained the lowest log reduction with HP (−1.54, (−4.80,1.72)). EW resulted in the highest log reduction in the three statuses.

We divided the bacteria into three statuses that are commonly found in the food industry (plankton, adhesion, and biofilm), which allowed us to more thoroughly understand the resistance of *L. monocytogenes* to sanitizers. Previous studies on the response of bacteria to sanitizers focused more on specific disinfectants [34], different bacteria [21], and environmental conditions [22]. Our study demonstrated *L. monocytogenes*’ high sensitivity to EW regardless of its status, which is consistent with the research performed by George et al. [22]. The use of EW to inactivate microorganisms and suppress their development to decrease postharvest loss and increase the shelf life of fresh fruits and vegetables during storage is the subject of an increasing number of studies. In addition, EW has also been applied to animal food and food contact surfaces [10]. In our metamodel, we considered the EW concentration (the available chlorine concentration, ACC) as well as the influence of environmental factors (treatment time and temperature), and for EW, the ORP (oxidation reduction potential) characteristics also had an impact on microorganism suppression. To be consistent with other studies exploring chemical disinfectants, we did not consider ORP characteristics. The high ORP properties of EW alter electron flow in microbial cells, disrupt cell membranes, oxidizes enzymes in cells, and lead to microbial cell death [35]. This seemed to imply the sensitivity of *L. monocytogenes* to EW. Thus, other characteristics of EW should be quantitatively studied in future research. Commonly used sanitizers are generally accepted to be efficient against *L. monocytogenes* in suspension; nevertheless, cells attached to surfaces may be more resistant to sanitizers than cells in suspension [30]. This statement is consistent with the effect of PAA, SH, and AA on *L. monocytogenes*. However, we were unable to find evidence supporting this assertion for all sanitizers. Several authors contend that a biofilm that has developed over a long period of time is more resistant to antimicrobial agents [36,37], whereas others have claimed that the biofilm’s age does not increase its susceptibility to disinfectants [38]. To understand this problem, supplementing the inactivation data of different biofilm periods is necessary, because the existing inactivation data and inactivation parameters are lacking.

### 3.4. Effect Size Estimation by Matrix

**Figure 4:**
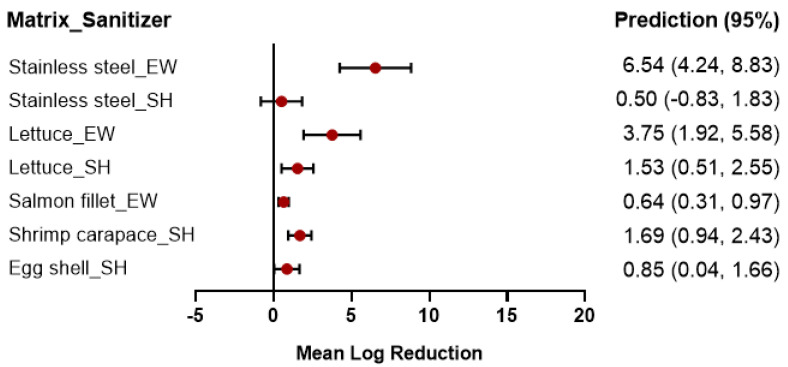
Forest plot of the predicted log reduction in *L. monocytogenes* on different matrices treated by 0.01% SH at 25 °C, 1 min, or 0.01% EW at 25 °C, 15 min.

The effect of sanitizers on *L. monocytogenes* has been observed by numerous studies, mostly focusing on three systems: liquid solutions [39,40], foods [41,42], and food contact surfaces [43,44,45]. According to the results of this meta-analysis, *L. monocytogenes* may differ in terms of their inactivation depending on the matrix. Different matrices with various physical and chemical characteristics may change the treatment efficiency. Based on this, we conducted models by considering different contact surfaces and examining the log reduction on food and food contact surfaces under hypothetical conditions. Nevertheless, the discontinuity of the available data hampered our work on data analysis via a matrix. Some fixed-effect levels were not sufficient to build a model, so some of the surfaces presented in Table 3 did not fully contain the parameters of the three fixed effects (Con, Temp, Time). By comparing the intercept (*β_0_*) results, we found that the two sanitizers (SH, EW) were more effective when used on stainless steel surfaces as opposed to other surfaces, as shown in Table 3. *L. monocytogenes* was less resistant to EW when on stainless steel than when on salmon fillets and lettuce (Table 3). Additionally, we again used the forest plot to compare the predicted log reduction of *L. monocytogenes* in different matrices under a hypothetical treatment with 0.01% SH or EW. Figure 4 illustrates that, when EW was the sanitizer used, the mean log reductions varied from 0.64 for the salmon fillet to 6.54 for stainless steel, whereas the log reduction varied from 0.50 to 1.69 for SH (Figure 4). *L. monocytogenes* had different resistance levels to different sanitizers on the same surface: the EW resistance of *L. monocytogenes* was lower on stainless steel, and the SH resistance of *L. monocytogenes* was higher on stainless steel. 

Because bacteria naturally have a predisposition to attach to surfaces as a survival mechanism, researchers have described the colonization of solid surfaces by bacteria as a fundamental and natural bacterial approach in a range of environments [46]. Researchers have frequently discovered *L. monocytogenes* in biofilm forms in food-processing facilities [47]. We found that identical sanitizing processes produced a different log reduction depending on the matrix type. We previously verified the effectiveness of EW (Table 1 and Table 2 and Figure 3). *L. monocytogenes* attaches to these substances differently depending on the matrix, which makes sanitizing *L. monocytogenes* on various matrices harder or easier. Salmon tissues are tough for disinfectants to reach and can harbor *L. monocytogenes*. Microorganisms on fresh produce cannot be eliminated by sanitizers because of the structure of the plant surface, including its nooks, crannies, and minute fissures, as well as the hydrophobic qualities of the waxy cuticle [48,49]. Additionally, chlorine reacts with organic matter on vegetables and reduces the effectiveness of chlorine [50].

## 4. Conclusions

Based on the meta-analysis approach, we quantitatively described and compared the impact of multiple factors on *L. monocytogenes* reduction using mixed-effects models with 896 extracted data. The hierarchical analysis supported the idea that the sanitizer type and concentration, as well as the treatment temperature and time, could primarily affect the survival of *L. monocytogenes*. *L. monocytogenes* generally exhibited higher resistance to citric acid and sodium hypochlorite. Meanwhile, *L. monocytogenes* in the adherent and biofilm status were more resistant to the investigated sanitizers. Besides, the physical and chemical properties of the foods and food contact surfaces might influence the sanitizer’s effectiveness. However, the research heterogeneity in strains and conditions also restricted the applicability of the established models and further inference on the trends of *L. monocytogenes* resistance. Therefore, future empirical study should systemically determine *L. monocytogenes* resistance to sanitizer by considering the strain variability and comprehensive environmental effects.

## Figures and Tables

**Table 1:** Estimated parameters of the mixed-effects models by sanitizer.

Sanitizer	Parameter	Mean	SE	*p*	AIC	BIC
CA	Intercept (*β_0_*)	2.93	0.76	<0.05	48.21	52.05
	Con (*β_1_*)	1.17	0.32	<0.05	*I²* = 96.20%	
	Time (*β_2_*)	0.09	0.02	<0.05		
	Temp (*β_3_*)	0.01	0.01	^1^ NS		
	Intercept (*ϑ*)	1.25				
	Residual (*ε*)	0.09				
CDS	Intercept (*β_0_*)	10.00	3.40	<0.05	180.80	192.50
	Con (*β_1_*)	2.13	0.50	<0.05	*I²* = 99.30%	
	Time (*β_2_*)	0.06	0.02	<0.05		
	Temp (*β_3_*)	0.14	0.04	<0.05		
	Intercept (*ϑ*)	4.6				
	Residual (*ε*)	0.44				
PAA	Intercept (*β_0_*)	7.49	1.21	<0.05	330.50	345.80
	Con (*β_1_*)	1.34	0.29	<0.05	*I²* = 99.40%	
	Time (*β_2_*)	0.14	0.03	<0.05		
	Temp (*β_3_*)	0.02	0.01	NS		
	Intercept (*ϑ*)	0.95				
	Residual (*ε*)	0.54				
EW	Intercept (*β_0_*)	11.63	1.53	<0.05	601.70	620.50
	Con (*β_1_*)	2.06	0.31	<0.05	*I²* = 99.80%	
	Time (*β_2_*)	0.02	0.01	NS		
	Temp (*β_3_*)	0.04	0.06	<0.05		
	Intercept (*ϑ*)	2.47				
	Residual (*ε*)	0.59				
SH	Intercept (*β_0_*)	4.63	0.90	<0.05	385.00	402.20
	Con (*β_1_*)	0.85	0.21	<0.05	*I²* = 99.40%	
	Time (*β_2_*)	0.08	0.01	<0.05		
	Temp (*β_3_*)	0.03	0.01	<0.05		
	Intercept (*ϑ*)	1.62				
	Residual (*ε*)	0.39				
CH	Intercept (*β_0_*)	52.95	6.04	<0.05	59.24	62.63
	Con (*β_1_*)	1.75	0.14	<0.05	*I²* = 99.60%	
	Time (*β_2_*)	−0.06	0.03	NS		
	Temp (*β_3_*)	−2	0.27	<0.05		
	Intercept (ϑ)	0.00				
	Residual (*ε*)	0.49				

^1^ NS: not significant.

**Table 2:** Estimated parameters of the mixed-effects models by *L. monocytogenes* biofilm status.

Status	Sanitizer	Parameter	Mean	SE	*p*	AIC	BIC
Plankton	PAA	Intercept (*β_0_*)	12.28	3.12	<0.05	170.50	180.12
		Con (*β_1_*)	2.46	0.68	<0.05	*I²* = 99.30%	
		Time (*β_2_*)	0.16	0.04	<0.05		
		Temp (*β_3_*)	0.03	0.02	^1^ NS		
		Intercept (*ϑ*)	2.72				
		Residual (*ε*)	0.64				
	SH	Intercept (*β_0_*)	7.02	6.02	NS	53.73	55.54
		Con (*β_1_*)	0.30	1.53	NS	*I²* = 97.30%	
		Time (*β_2_*)	−0.02	0.03	NS		
		Temp (*β_3_*)	0.03	0.02	NS		
		Intercept (*ϑ*)	1.38				
		Residual (*ε*)	0.39				
	HP	Intercept (*β_0_*)	12.01	6.96	NS	135.90	146.77
		Con (*β_1_*)	7.50	3.6	<0.05	*I²* = 93.00%	
		Time (*β_2_*)	0.09	0.04	<0.05		
		Temp (*β_3_*)	0.15	0.19	NS		
		Intercept (*ϑ*)	0				
		Residual (*ε*)	0.46				
	AA	Intercept (*β_0_*)	12.47	6.12	NS	30.10	22.25
		Con (*β_1_*)	9.77	4.44	NS	*I²* = 98.40%	
		Time (*β_2_*)	0.22	0.12	NS		
		Temp (*β_3_*)	0.11	0.04	NS		
		Intercept (*ϑ*)	0.59				
		Residual (*ε*)	0.54				
	CDS	Intercept (*β_0_*)	10.21	5.89	NS	101.40	108.92
		Con (*β_1_*)	2.24	0.58	<0.05	*I²* = 99.40%	
		Time (*β_2_*)	0.06	0.03	<0.05		
		Temp (*β_3_*)	0.16	0.05	<0.05		
		Intercept (*ϑ*)	8.01				
		Residual (*ε*)	0.45				
	EW	Intercept (*β_0_*)	14.83	2.44	<0.05	276.60	289.98
		Con (*β_1_*)	2.24	0.48	<0.05	*I²* = 99.90%	
		Time (*β_2_*)	0.11	0.1	NS		
		Temp (*β_3_*)	0.05	0.02	NS		
		Intercept (*ϑ*)	1.44				
		Residual (*ε*)	0.74				
Adhesion	PAA	Intercept (*β_0_*)	8.25	3.92	NS	73.99	78.99
		Con (*β_1_*)	1.25	0.81	NS	*I²* = 98.40%	
		Time (*β_2_*)	0.03	0.15	NS		
		Temp (*β_3_*)	−0.02	0.05	NS		
		Intercept (*ϑ*)	0.83				
		Residual (*ε*)	0.46				
	SH	Intercept (*β_0_*)	2.26	0.83	<0.05	170.8	182.41
		Con (*β_1_*)	0.33	0.19	NS	*I²* = 96.40%	
		Time (*β_2_*)	0.06	0.02	<0.05		
		Temp (*β_3_*)	0.03	0.02	NS		
		Intercept (*ϑ*)	0.71				
		Residual (*ε*)	0.44				
	AA	Intercept (*β_0_*)	4.65	0.74	<0.05	18.72	20.54
		Con (*β_1_*)	0.47	0.17	<0.05	*I²* = 96.50%	
		Time (*β_2_*)	0.05	0.01	<0.05		
		Temp (*β_3_*)	−0.15	0.04	<0.05		
		Intercept (*ϑ*)	0.39				
		Residual (*ε*)	0.05				
	CA	Intercept (*β_0_*)	3.27	0.78	<0.05	40.05	45.96
		Con (*β_1_*)	1.22	0.32	<0.05	*I²* = 93.00%	
		Time (*β_2_*)	0.07	0.02	<0.05		
		Temp (*β_3_*)	0.01	0.01	NS		
		Intercept (*ϑ*)	1.21				
		Residual (*ε*)	0.08				
	EW	Intercept (*β_0_*)	11.75	1.64	<0.05	279.90	294.85
		Con (*β_1_*)	2.33	0.36	<0.05	*I²* = 99.00%	
		Time (*β_2_*)	0.01	0.01	NS		
		Temp (*β_3_*)	0.03	0.02	NS		
		Intercept (*ϑ*)	1.47				
		Residual (*ε*)	0.45				
	LA	Intercept (*β_0_*)	5.69	1.52	<0.05	40.52	43.43
		Con (*β_1_*)	1.08	0.26	<0.05	*I²* = 98.10%	
		Time (*β_2_*)	0.01	0.01	NS		
		Temp (*β_3_*)	−0.09	0.07	NS		
		Intercept (*ϑ*)	0.82				
		Residual (*ε*)	0.12				
Biofilm	PAA	Intercept (*β_0_*)	7.01	10	NS	101.60	110.40
		Con (*β_1_*)	0.09	0.57	NS	*I²* = 99.80%	
		Time (*β_2_*)	0.14	0.1	NS		
		Temp (*β_3_*)	−0.15	0.4	NS		
		Intercept (*ϑ*)	0				
		Residual (*ε*)	0.48				
	SH	Intercept (*β_0_*)	6.22	0.86	<0.05	152.30	164.94
		Con (*β_1_*)	1.39	0.23	<0.05	*I²* = 98.60%	
		Time (*β_2_*)	0.11	0.01	<0.05		
		Temp (*β_3_*)	0.01	0.01	NS		
		Intercept (*ϑ*)	0.98				
		Residual (*ε*)	0.28				
	HP	Intercept (*β_0_*)	30.7	15.8	NS	25.73	27.58
		Con (*β_1_*)	0.84	0.36	<0.05	*I²* = 98.90%	
		Time (*β_2_*)	0.19	0.05	<0.05		
		Temp (*β_3_*)	−1.33	0.73	NS		
		Intercept (*ϑ*)	1.33				
		Residual (*ε*)	0.15				
	EW	Intercept (*β_0_*)	98.1	20.6	<0.05	18.83	15.15
		Con (*β_1_*)	0.39	0.67	NS	*I²* = 99.60%	
		Time (*β_2_*)	0.48	0.35	NS		
		Temp (*β_3_*)	−3.84	0.92	NS		
		Intercept (*ϑ*)	0.32				
		Residual (*ε*)	0.24				
	LA	Intercept (*β_0_*)	12.87	13.5	NS	36.60	34.26
		Con (*β_1_*)	1.94	0.81	NS	*I²* = 88.60%	
		Time (*β_2_*)	0.00	0.00	NS		
		Temp (*β_3_*)	−0.32	0.61	NS		
		Intercept (*ϑ*)	2.45				
		Residual (*ε*)	0.19				

^1^ NS: not significant.

**Table 3:** Estimated parameters of the mixed-effects models by matrix.

Matrix	Sanitizer	Parameter	Mean	SE	*p*	AIC	BIC
Stainless steel	SH	Intercept (*β_0_*)	37.58	14.75	<0.05	96.80	105.95
		Con (*β_1_*)	2.21	0.32	<0.05	*I²* = 98.60%	
		Time (*β_2_*)	0.29	0.05	<0.05		
		Temp (*β_3_*)	−1.27	0.64	^1^ NS		
		Intercept (*ϑ*)	1.48				
		Residual (*ε*)	0.27				
	EW	Intercept (*β_0_*)	23.81	22.77	NS	99.27	109.10
		Con (*β_1_*)	3.72	0.42	<0.05	*I²* = 99.70%	
		Time (*β_2_*)	0.28	0.048	<0.05		
		Temp (*β_3_*)	−0.25	0.94	NS		
		Intercept (*ϑ*)	2.72				
		Residual (*ε*)	0.64				
Lettuce	SH	Intercept (*β_0_*)	3.21	0.46	<0.05	20.07	22.06
		Con (*β_1_*)	0.42	0.08	<0.05	*I²* = 93.30%	
		Time (*β_2_*)	0.01	0.01	NS		
		Temp (*β_3_*)	^2^ -				
		Intercept (*ϑ*)	0.41				
		Residual (*ε*)	0.11				
	EW	Intercept (*β_0_*)	5.67	1.57	<0.05	55.83	62.37
		Con (*β_1_*)	0.83	0.33	<0.05	*I²* = 95.20%	
		Time (*β_2_*)	0.06	0.05	NS		
		Temp (*β_3_*)	0.03	0.01	<0.05		
		Intercept (*ϑ*)	1.52				
		Residual (*ε*)	0.17				
Egg shell	SH	Intercept (*β_0_*)	7.59	1.63	<0.05	6.73	1.50
		Con (*β_1_*)	1.69	0.41	NS	*I²* = 66.50%	
		Time (*β_2_*)	-				
		Temp (*β_3_*)	-				
		Intercept (*ϑ*)	0.14				
		Residual (*ε*)	0.11				
Shrimp carapace	SH	Intercept (*β_0_*)	0.96	0.18	<0.05	91.87	99.92
		Con (*β_1_*)	-			*I²* = 96.70%	
		Time (*β_2_*)	0.12	0.01	<0.05		
		Temp (*β_3_*)	0.03	0.01	<0.05		
		Intercept (*ϑ*)	2.72				
		Residual (*ε*)	0.64				
Salmon fillet	EW	Intercept (*β_0_*)	−0.1	0.07	NS	−0.45	0.54
		Con (*β_1_*)	-			*I²* = 0.00%	
		Time (*β_2_*)	0.01	0	<0.05		
		Temp (*β_3_*)	0.03	0	<0.05		
		Intercept (*ϑ*)	0.02				
		Residual (*ε*)	0.03				

^1^ NS: not significant.^2^ -: parameter not calculated.

## Supplementary Materials

The following are available online at https://www.mdpi.com/article/10.3390/foods12010154/s1, Table S1: The extracted data for meta-analysis. References [51,52,53,54,55,56,57,58,59,60,61,62,63,64,65,66,67,68,69,70,71,72,73,74,75,76,77,78,79,80,81,82,83,84,85,86,87,88,89,90,91,92,93,94,95,96,97,98,99,100,101,102,103,104,105,106,107,108,109,110,111,112,113,114,115,116,117,118,119,120,121,122] are cited in the supplementary materials.
