# Supplementary material for: A Meta-Analysis and Systematic Review of Listeria monocytogenes Response to Sanitizer Treatments
Source: Foods. 2022 Dec 28;12(1):154. doi: 10.3390/foods12010154 (PMC9818549; doi:10.3390/foods12010154)
Supplement: Supplementary file 1 [file foods-12-00154-s001.zip › foods-2056596-supplementary.pdf]

Table S1. The extracted data for meta-analysis.

Strain type	Biofilm status	Matrix	Sanitizer type	Sanitizer concentration	Treatment time	Temperature	Bacterial log-reduction	Standard deviation	Replication	Reference
ATCC 7644	Adhesion	Lettuce	LA	0.500000%	5.0	20.0	2.00	0.01	3	[51]
ATCC 7644	Adhesion	Lettuce	LA	1.000000%	5.0	20.0	2.20	0.01	3	
ATCC 7644	Adhesion	Lettuce	CA	0.500000%	5.0	20.0	1.50	0.03	3	
ATCC 7644	Adhesion	Lettuce	CA	1.000000%	2.0	20.0	1.60	0.03	3	
ATCC 7644	Adhesion	Lettuce	CA	1.000000%	5.0	20.0	1.80	0.03	3	
ATCC 7644	Adhesion	Lettuce	AA	0.500000%	5.0	20.0	1.30	0.03	3	
ATCC 7644	Adhesion	Lettuce	AA	1.000000%	5.0	20.0	1.40	0.03	3	
ATCC 7644	Adhesion	Lettuce	ASA	0.500000%	5.0	20.0	1.20	0.03	3	
ATCC 7644	Adhesion	Lettuce	ASA	1.000000%	5.0	20.0	1.30	0.03	3	
ATCC 7644	Adhesion	Lettuce	LA	1.000000%	2.0	20.0	2.10	0.05	3	
ATCC 7644	Adhesion	Lettuce	CA	0.500000%	2.0	20.0	1.40	0.10	3	
ATCC 7644	Adhesion	Lettuce	AA	0.500000%	2.0	20.0	1.20	0.10	3	
ATCC 7644	Adhesion	Lettuce	AA	1.000000%	2.0	20.0	1.30	0.10	3	
ATCC 7644	Adhesion	Lettuce	ASA	0.500000%	2.0	20.0	1.10	0.10	3	
ATCC 7644	Adhesion	Lettuce	ASA	1.000000%	2.0	20.0	1.30	0.10	3	
ATCC 7644	Adhesion	Lettuce	LA	0.500000%	2.0	20.0	2.00	0.11	3	
NCTC 11994	Adhesion	Chicken leg	CA	2.000000%	15.0	20.0	1.27	0.20	5	[52]
NCTC 11994	Adhesion	Chicken leg	CDS	0.005000%	15.0	20.0	0.31	0.24	5	
NCTC 11994	Adhesion	Chicken leg	ASP	0.120000%	15.0	20.0	1.03	0.27	5	
NCTC 11994	Adhesion	Chicken leg	TSP	12.000000%	15.0	20.0	1.11	0.34	5	
NCTC 11994	Adhesion	Chicken leg	TSP	12.000000%	15.0	50.0	1.46	0.44	5	
NCTC 11994	Adhesion	Chicken leg	CA	2.000000%	15.0	50.0	1.40	0.50	5	
NCTC 11994	Adhesion	Chicken leg	CA	2.000000%	15.0	4.0	0.70	0.52	5	
NCTC 11994	Adhesion	Chicken leg	ASP	0.120000%	15.0	50.0	0.57	0.73	5	
NCTC 11994	Adhesion	Chicken leg	ASP	0.120000%	15.0	4.0	0.44	0.81	5	
NCTC 11994	Adhesion	Chicken leg	TSP	12.000000%	15.0	4.0	0.34	0.84	5	
NCTC 11994	Adhesion	Chicken leg	CDS	0.005000%	15.0	4.0	0.06	0.84	5	

ATCC 7644	Adhesion	Polypropylene	PAA	0.200000%	10.0	25.0	4.20	0.00	3	[53]
ATCC 7644	Adhesion	Polypropylene	BG	0.500000%	10.0	25.0	2.70	0.10	3	
ATCC 7644	Adhesion	Polypropylene	BG	1.000000%	10.0	25.0	3.30	0.10	3	
ATCC 7644	Adhesion	Polypropylene	BG	0.200000%	10.0	25.0	1.70	0.10	3	
ATCC 7644	Adhesion	Polypropylene	BG	2.000000%	10.0	25.0	2.70	0.10	3	
ATCC 7644	Adhesion	Polypropylene	BG	0.200000%	10.0	25.0	1.10	0.10	3	
ATCC 7644	Adhesion	Polypropylene	BG	0.500000%	10.0	25.0	2.10	0.10	3	
ATCC 7644	Adhesion	Polypropylene	BG	2.000000%	10.0	25.0	2.80	0.10	3	
ATCC 7644	Adhesion	Polypropylene	BG	0.200000%	10.0	25.0	2.00	0.20	3	
ATCC 7644	Adhesion	Polypropylene	BG	0.500000%	10.0	25.0	2.40	0.20	3	
ATCC 7644	Adhesion	Polypropylene	BG	1.000000%	10.0	25.0	2.80	0.20	3	
ATCC 7644	Adhesion	Polypropylene	BG	1.000000%	10.0	25.0	2.50	0.20	3	
ATCC 7644	Adhesion	Polypropylene	BG	2.000000%	10.0	25.0	3.50	0.30	3	
ATCC 7644	Adhesion	Stainless steel	PAA	0.200000%	10.0	25.0	4.70	0.00	3	
ATCC 7644	Adhesion	Stainless steel	BG	0.200000%	10.0	25.0	0.60	0.10	3	
ATCC 7644	Adhesion	Stainless steel	BG	0.500000%	10.0	25.0	1.30	0.10	3	
ATCC 7644	Adhesion	Stainless steel	BG	1.000000%	10.0	25.0	3.20	0.10	3	
ATCC 7644	Adhesion	Stainless steel	BG	2.000000%	10.0	25.0	3.40	0.10	3	
ATCC 7644	Adhesion	Stainless steel	BG	0.200000%	10.0	25.0	0.20	0.10	3	
ATCC 7644	Adhesion	Stainless steel	BG	0.500000%	10.0	25.0	0.90	0.10	3	
ATCC 7644	Adhesion	Stainless steel	BG	1.000000%	10.0	25.0	1.90	0.10	3	
ATCC 7644	Adhesion	Stainless steel	BG	2.000000%	10.0	25.0	3.30	0.10	3	
ATCC 7644	Adhesion	Stainless steel	BG	0.200000%	10.0	25.0	1.00	0.10	3	
ATCC 7644	Adhesion	Stainless steel	BG	0.500000%	10.0	25.0	2.00	0.10	3	
ATCC 7644	Adhesion	Stainless steel	BG	1.000000%	10.0	25.0	2.70	0.10	3	
ATCC 7644	Adhesion	Stainless steel	BG	2.000000%	10.0	25.0	3.90	0.10	3	
Scott A	Adhesion	Cherry tomato	SB	0.300000%	3.0	21.0	4.80	Unclear	4	[54]
Dairy isolated	Adhesion	Tomato	EW	0.009210%	1.0	23.0	4.80	0.37	4	[55]
Dairy isolated	Adhesion	Tomato	EW	0.009210%	0.5	23.0	4.61	0.61	4	
ATCC 19115	Adhesion	Oyster mushroom	EW	0.005000%	3.0	23.0	2.08	0.04	3	[56]
ATCC 19115	Adhesion	Oyster mushroom	CA	1.000000%	3.0	23.0	1.42	0.06	2	
ATCC 19115	Adhesion	Oyster mushroom	OW	0.000500%	3.0	23.0	1.06	0.06	2	

ATCC 19115	Adhesion	Oyster mushroom	EW	0.000500%	3.0	23.0	2.16	0.06	3	
ATCC 19115	Adhesion	Oyster mushroom	SH	0.010000%	3.0	23.0	1.94	0.06	2	
Cocktail	Adhesion	Lettuce	EW	0.003000%	3.0	15.0	1.39	Unclear	Unclear	[57]
Cocktail	Adhesion	Lettuce	EW	0.003000%	3.0	35.0	1.77	Unclear	Unclear	
Cocktail	Adhesion	Lettuce	EW	0.007000%	3.0	15.0	2.01	Unclear	Unclear	
Cocktail	Adhesion	Lettuce	EW	0.007000%	3.0	35.0	2.79	Unclear	Unclear	
Cocktail	Adhesion	Lettuce	EW	0.005000%	1.0	15.0	1.88	Unclear	Unclear	
Cocktail	Adhesion	Lettuce	EW	0.005000%	1.0	35.0	2.17	Unclear	Unclear	
Cocktail	Adhesion	Lettuce	EW	0.005000%	5.0	15.0	2.04	Unclear	Unclear	
Cocktail	Adhesion	Lettuce	EW	0.005000%	5.0	35.0	2.58	Unclear	Unclear	
Cocktail	Adhesion	Lettuce	EW	0.003000%	1.0	25.0	1.52	Unclear	Unclear	
Cocktail	Adhesion	Lettuce	EW	0.007000%	1.0	25.0	2.53	Unclear	Unclear	
Cocktail	Adhesion	Lettuce	EW	0.003000%	5.0	25.0	1.71	Unclear	Unclear	
Cocktail	Adhesion	Lettuce	EW	0.007000%	5.0	25.0	2.73	Unclear	Unclear	
Cocktail	Adhesion	Lettuce	EW	0.005000%	3.0	25.0	2.21	Unclear	Unclear	
Cocktail	Adhesion	Lettuce	EW	0.005000%	3.0	25.0	2.25	Unclear	Unclear	
Cocktail	Adhesion	Lettuce	EW	0.005000%	3.0	25.0	2.18	Unclear	Unclear	
Cocktail	Adhesion	Lettuce	EW	0.005000%	3.0	25.0	2.27	Unclear	Unclear	
Cocktail	Adhesion	Lettuce	EW	0.005000%	3.0	25.0	2.31	Unclear	Unclear	
CECT 936	Adhesion	Lettuce	SH	0.000070%	5.0	22.5	1.05	0.05	3	[58]
CECT 936	Adhesion	Lettuce	SH	0.000800%	5.0	22.5	1.68	0.06	3	
CECT 936	Adhesion	Lettuce	SH	0.000070%	30.0	22.5	1.11	0.07	3	
CECT 936	Adhesion	Lettuce	SH	0.000400%	30.0	22.5	1.91	0.08	3	
CECT 936	Adhesion	Lettuce	SH	0.000400%	5.0	22.5	1.57	0.09	3	
CECT 936	Adhesion	Lettuce	SH	0.000400%	15.0	22.5	1.31	0.10	3	
CECT 936	Adhesion	Lettuce	SH	0.004000%	30.0	22.5	1.97	0.10	3	
CECT 936	Adhesion	Lettuce	SH	0.000070%	15.0	22.5	1.13	0.12	3	
CECT 936	Adhesion	Lettuce	SH	0.004000%	5.0	22.5	1.72	0.14	3	
CECT 936	Adhesion	Lettuce	SH	0.000800%	15.0	22.5	1.72	0.17	3	
CECT 936	Adhesion	Lettuce	SH	0.000800%	30.0	22.5	2.04	0.20	3	
CECT 936	Adhesion	Lettuce	SH	0.004000%	15.0	22.5	1.87	0.20	3	
Cocktail	Adhesion	Chinese cabbage	EW	0.002200%	3.0	23.0	1.19	0.08	5	[59]

Cocktail	Adhesion	Lettuce	EW	0.002200%	3.0	23.0	1.20	0.24	5	
Cocktail	Adhesion	Sesame leaf	EW	0.002200%	3.0	23.0	1.31	0.38	5	
Cocktail	Adhesion	Spinach	EW	0.002200%	3.0	23.0	1.48	0.33	5	
Cocktail	Adhesion	Grape tomato	SH	0.004900%	5.0	22.0	2.92	0.38	3	[60]
Scott A	Adhesion	Cabbage	SH	10.000000%	5.0	22.5	2.50	Unclear	3	[61]
Scott A	Adhesion	Sprout	SH	10.000000%	5.0	22.5	2.10	Unclear	3	
Cocktail	Adhesion	Spinach	CA	1.000000%	3.7	22.0	0.57	0.26	3	[62]
Cocktail	Adhesion	Spinach	CA	1.000000%	7.4	22.0	1.13	0.28	3	
Cocktail	Adhesion	Spinach	CA	1.000000%	11.1	22.0	1.23	0.33	3	
Cocktail	Adhesion	Dates	PAA	0.007500%	1.0	22.5	4.96	0.18	2	[63]
Cocktail	Adhesion	Dates	PAA	0.007500%	0.5	22.5	3.91	0.28	2	
Cocktail	Adhesion	Dates	PAA	0.007500%	0.0	22.5	2.40	0.35	2	
ATCC 19111	Adhesion	Tatsoi baby leaf	LA	0.250000%	5.0	22.5	1.69	0.02	3	[64]
ATCC 19111	Adhesion	Tatsoi baby leaf	LA	0.500000%	5.0	22.5	2.26	0.12	3	
ATCC 19111	Adhesion	Tatsoi baby leaf	LA	2.000000%	5.0	22.5	2.99	0.17	3	
ATCC 19111	Adhesion	Tatsoi baby leaf	LA	1.000000%	5.0	22.5	2.83	0.20	3	
Cocktail	Adhesion	Broccoli	MA	2.000000%	5.0	22.0	1.30	Unclear	3	[65]
Cocktail	Adhesion	Kale	SH	0.010000%	3.0	22.5	3.76	0.26	3	[66]
Cocktail	Adhesion	Lettuce	SH	0.010000%	3.0	22.5	1.40	0.13	3	
Cocktail	Adhesion	Spinach	SH	0.010000%	3.0	22.5	1.91	0.12	3	
Cocktail	Adhesion	Kale	BAC	0.000500%	3.0	22.5	0.97	0.06	3	[67]
Cocktail	Adhesion	Kale	CPA	0.000500%	3.0	22.5	1.22	0.06	3	
Cocktail	Adhesion	Kale	T80	0.000500%	3.0	22.5	0.77	0.08	3	
Cocktail	Adhesion	Kale	SH	0.005000%	3.0	22.5	1.23	0.11	3	
Cocktail	Adhesion	Kale	SDS	0.000500%	3.0	22.5	1.09	0.11	3	
Cocktail	Adhesion	Lettuce	BEC	0.010000%	3.0	22.5	1.18	Unclear	2	[13]
Cocktail	Adhesion	Lettuce	BEC	0.020000%	3.0	22.5	1.52	Unclear	2	
Cocktail	Adhesion	Lettuce	BEC	0.030000%	3.0	22.5	1.84	Unclear	2	
Cocktail	Adhesion	Lettuce	BEC	0.050000%	3.0	22.5	1.95	Unclear	2	
Cocktail	Adhesion	Glazed ceramic tile	EW	0.005000%	5.0	22.5	4.24	Unclear	Unclear	[68]
Cocktail	Adhesion	Stainless steel	EW	0.004000%	5.0	22.5	1.55	0.26	Unclear	
Cocktail	Adhesion	Stainless steel	EW	0.005000%	5.0	22.5	3.37	Unclear	Unclear	

Cocktail	Adhesion	Unglazed quarry floor tile	EW	0.005000%	5.0	22.5	5.12	Unclear	Unclear	
BAA-679	Adhesion	Sprout	SH	0.017000%	3.0	22.5	1.30	0.30	3	[69]
BAA-679	Adhesion	Sprout	PAA	0.007000%	3.0	22.5	1.80	0.40	3	
BAA-679	Adhesion	Sprout	PAA	0.002500%	1.5	22.5	1.00	Unclear	3	
BAA-679	Adhesion	Sprout	SH	0.010600%	1.5	22.5	0.70	Unclear	3	
Cocktail	Adhesion	Sprout	FA	0.500000%	3.0	22.5	2.17	Unclear	3	[70]
Scott A	Adhesion	Shrimp carapace	SH	0.010000%	5.0	4.0	2.31	0.00	3	[43]
V7	Adhesion	Shrimp carapace	SH	0.010000%	5.0	4.0	2.64	0.00	3	
V7	Adhesion	Shrimp carapace	SH	0.010000%	10.0	4.0	2.64	0.00	3	
Scott A	Adhesion	Shrimp carapace	SH	0.010000%	0.5	25.0	1.11	0.07	3	
V7	Adhesion	Shrimp carapace	SH	0.010000%	3.0	4.0	0.77	0.13	3	
Scott A	Adhesion	Shrimp carapace	SH	0.010000%	20.0	4.0	3.17	0.17	3	
V7	Adhesion	Shrimp carapace	SH	0.010000%	5.0	25.0	2.34	0.18	3	
V7	Adhesion	Shrimp carapace	SH	0.010000%	0.5	4.0	0.67	0.19	3	
V7	Adhesion	Shrimp carapace	SH	0.010000%	20.0	4.0	3.27	0.20	3	
V7	Adhesion	Shrimp carapace	SH	0.010000%	10.0	25.0	3.01	0.20	3	
Scott A	Adhesion	Shrimp carapace	SH	0.010000%	10.0	25.0	3.49	0.23	3	
Scott A	Adhesion	Shrimp carapace	SH	0.010000%	20.0	25.0	4.72	0.24	3	
Scott A	Adhesion	Shrimp carapace	SH	0.010000%	3.0	25.0	2.39	0.30	3	
Scott A	Adhesion	Shrimp carapace	SH	0.010000%	5.0	25.0	3.05	0.33	3	
Scott A	Adhesion	Shrimp carapace	SH	0.010000%	10.0	4.0	2.68	0.36	3	
V7	Adhesion	Shrimp carapace	SH	0.010000%	20.0	25.0	3.88	0.37	3	
V7	Adhesion	Shrimp carapace	SH	0.010000%	0.5	25.0	1.17	0.64	3	
Scott A	Adhesion	Shrimp carapace	SH	0.010000%	3.0	4.0	0.95	0.68	3	
V7	Adhesion	Shrimp carapace	SH	0.010000%	3.0	25.0	2.46	0.83	3	
Scott A	Adhesion	Shrimp carapace	SH	0.010000%	0.5	4.0	0.30	0.95	3	
Cocktail	Adhesion	Strawberry	PAA	0.004000%	2.0	10.0	3.80	0.00	6	[41]
Cocktail	Adhesion	Strawberry	PAA	0.008000%	2.0	10.0	3.80	0.00	6	
Cocktail	Adhesion	Strawberry	PAA	0.012000%	2.0	10.0	3.80	0.00	6	
Cocktail	Adhesion	Strawberry	LA	2.500000%	2.0	10.0	3.00	0.00	6	
Cocktail	Adhesion	Strawberry	SH	0.010000%	2.0	10.0	2.40	0.10	6	

Cocktail	Adhesion	Strawberry	CA	1.000000%	2.0	10.0	3.20	0.20	6	
Cocktail	Adhesion	Strawberry	SH	0.010000%	2.0	10.0	3.20	0.30	6	
Cocktail	Adhesion	Strawberry	SH	0.010000%	2.0	10.0	2.90	0.40	6	
Cocktail	Adhesion	Strawberry	SH	0.010000%	2.0	10.0	1.30	0.40	6	
Cocktail	Adhesion	Strawberry	SH	0.010000%	2.0	10.0	2.70	0.40	6	
Cocktail	Adhesion	Strawberry	LA	1.000000%	2.0	10.0	2.70	0.60	6	
Cocktail	Adhesion	Strawberry	AA	1.000000%	2.0	10.0	2.50	0.60	6	
Cocktail	Adhesion	Strawberry	AA	2.500000%	2.0	10.0	2.50	0.60	6	
Cocktail	Adhesion	Strawberry	AA	5.000000%	2.0	10.0	2.80	0.60	6	
Cocktail	Adhesion	Strawberry	CA	2.500000%	2.0	10.0	4.00	0.60	6	
Cocktail	Adhesion	Strawberry	CA	5.000000%	2.0	10.0	4.20	0.60	6	
Cocktail	Adhesion	Strawberry	HP	2.500000%	2.0	10.0	3.80	0.70	6	
Cocktail	Adhesion	Strawberry	HP	5.000000%	2.0	10.0	5.40	0.80	6	
Cocktail	Adhesion	Strawberry	HP	1.000000%	2.0	10.0	2.40	0.90	6	
Cocktail	Adhesion	Strawberry	LA	5.000000%	2.0	10.0	2.70	0.90	6	
Scott A	Adhesion	Salmon fillet	EW	0.008300%	2.0	22.0	0.40	Unclear	Unclear	[71]
Scott A	Adhesion	Salmon fillet	EW	0.008300%	4.0	22.0	0.48	Unclear	Unclear	
Scott A	Adhesion	Salmon fillet	EW	0.008300%	8.0	22.0	0.52	Unclear	Unclear	
Scott A	Adhesion	Salmon fillet	EW	0.008300%	16.0	22.0	0.58	Unclear	Unclear	
Scott A	Adhesion	Salmon fillet	EW	0.008300%	32.0	22.0	0.74	Unclear	Unclear	
Scott A	Adhesion	Salmon fillet	EW	0.008300%	64.0	22.0	0.86	Unclear	Unclear	
Scott A	Adhesion	Salmon fillet	EW	0.008300%	2.0	35.0	0.79	Unclear	Unclear	
Scott A	Adhesion	Salmon fillet	EW	0.008300%	4.0	35.0	0.88	Unclear	Unclear	
Scott A	Adhesion	Salmon fillet	EW	0.008300%	8.0	35.0	0.90	Unclear	Unclear	
Scott A	Adhesion	Salmon fillet	EW	0.008300%	16.0	35.0	0.93	Unclear	Unclear	
Scott A	Adhesion	Salmon fillet	EW	0.008300%	32.0	35.0	0.99	Unclear	Unclear	
Scott A	Adhesion	Salmon fillet	EW	0.008300%	64.0	35.0	1.12	Unclear	Unclear	
Cocktail	Adhesion	Lettuce	EW	0.004500%	1.0	22.0	5.09	Unclear	9	[72]
Cocktail	Adhesion	Lettuce	EW	0.004500%	3.0	22.0	5.55	Unclear	9	
Cocktail	Adhesion	Stainless steel	EW	0.010000%	1.0	25.0	2.28	0.00	2	[73]
Cocktail	Adhesion	Stainless steel	EW	0.015300%	3.0	25.0	3.02	0.00	2	
Cocktail	Adhesion	Stainless steel	EW	0.010300%	3.0	25.0	3.15	0.01	2	

Cocktail	Adhesion	Stainless steel	EW	0.015400%	1.0	25.0	2.66	0.04	2	
Cocktail	Adhesion	Stainless steel	EW	0.005000%	1.0	25.0	1.06	0.07	2	
Cocktail	Adhesion	Stainless steel	EW	0.005000%	3.0	25.0	1.73	0.07	2	
Cocktail	Adhesion	Stainless steel	EW	0.020700%	0.5	25.0	2.28	0.07	2	
Cocktail	Adhesion	Stainless steel	EW	0.020700%	1.0	25.0	3.38	0.08	2	
Cocktail	Adhesion	Stainless steel	EW	0.005400%	1.0	25.0	1.07	0.10	2	
Cocktail	Adhesion	Stainless steel	EW	0.020700%	3.0	25.0	4.34	0.10	2	
Cocktail	Adhesion	Stainless steel	EW	0.020700%	6.0	25.0	5.64	0.11	2	
Cocktail	Adhesion	Stainless steel	EW	0.005400%	6.0	25.0	1.55	0.11	2	
Cocktail	Adhesion	Stainless steel	EW	0.020700%	6.0	25.0	5.22	0.12	2	
Cocktail	Adhesion	Stainless steel	EW	0.010300%	6.0	25.0	3.14	0.13	2	
Cocktail	Adhesion	Stainless steel	EW	0.005000%	0.5	25.0	1.04	0.16	2	
Cocktail	Adhesion	Stainless steel	EW	0.020700%	0.5	25.0	2.28	0.16	2	
Cocktail	Adhesion	Stainless steel	EW	0.015400%	0.5	25.0	2.16	0.18	2	
Cocktail	Adhesion	Stainless steel	EW	0.015400%	6.0	25.0	4.02	0.18	2	
Cocktail	Adhesion	Stainless steel	EW	0.015300%	0.5	25.0	1.56	0.20	2	
Cocktail	Adhesion	Stainless steel	EW	0.010000%	0.5	25.0	1.51	0.22	2	
Cocktail	Adhesion	Stainless steel	EW	0.015300%	6.0	25.0	3.32	0.22	2	
Cocktail	Adhesion	Stainless steel	EW	0.015400%	3.0	25.0	3.35	0.23	2	
Cocktail	Adhesion	Stainless steel	EW	0.010300%	1.0	25.0	2.56	0.27	2	
Cocktail	Adhesion	Stainless steel	EW	0.005400%	3.0	25.0	1.32	0.27	2	
Cocktail	Adhesion	Stainless steel	EW	0.010000%	6.0	25.0	2.07	0.27	2	
Cocktail	Adhesion	Stainless steel	EW	0.005400%	0.5	25.0	0.92	0.28	2	
Cocktail	Adhesion	Stainless steel	EW	0.015300%	1.0	25.0	2.19	0.32	2	
Cocktail	Adhesion	Stainless steel	EW	0.020700%	1.0	25.0	3.06	0.34	2	
Cocktail	Adhesion	Stainless steel	EW	0.010000%	3.0	25.0	2.07	0.37	2	
Cocktail	Adhesion	Stainless steel	EW	0.005000%	6.0	25.0	2.30	0.60	2	
Cocktail	Adhesion	Stainless steel	EW	0.020700%	3.0	25.0	4.35	0.61	2	
Cocktail	Adhesion	Stainless steel	EW	0.010300%	0.5	25.0	2.12	0.79	2	
Cocktail	Adhesion	Lettuce	SHD	2.000000%	5.0	4.0	2.10	0.00	3	[74]
Cocktail	Adhesion	Lettuce	PAA	0.007800%	5.0	4.0	2.70	0.10	3	
Cocktail	Adhesion	Lettuce	EW	0.010000%	5.0	20.0	3.00	0.20	3	

Cocktail	Adhesion	Lettuce	EW	0.010000%	5.0	4.0	2.50	0.30	3	
Cocktail	Adhesion	Lettuce	SHD	2.000000%	5.0	20.0	1.80	0.40	3	
Cocktail	Adhesion	Lettuce	PAA	0.007800%	5.0	20.0	3.10	0.50	3	
ATCC 19115	Adhesion	Lettuce	EW	0.000520%	1.0	35.0	3.76	Unclear	Unclear	[75]
ATCC 19115	Adhesion	Lettuce	EW	0.005100%	1.0	35.0	3.68	Unclear	Unclear	
Cocktail	Adhesion	Spinach	OW	0.000500%	3.0	23.0	1.40	Unclear	3	[76]
Cocktail	Adhesion	Spinach	CA	1.000000%	3.0	23.0	1.70	Unclear	3	
Cocktail	Adhesion	Spinach	SH	0.010000%	3.0	23.0	2.20	Unclear	3	
Cocktail	Adhesion	Spinach	EW	0.000500%	3.0	23.0	0.95	Unclear	Unclear	
Cocktail	Adhesion	Spinach	EW	0.005000%	3.0	23.0	2.80	Unclear	Unclear	
Cocktail	Adhesion	Lettuce	AA	1.174000%	15.0	20.0	1.13	0.00	3	[77]
Cocktail	Adhesion	Lettuce	AA	0.880500%	15.0	20.0	0.86	0.02	3	
ATCC 19115	Adhesion	Mango	AA	0.500000%	5.0	22.5	0.80	0.20	2	[78]
ATCC 19115	Adhesion	Mango	SH	0.020000%	5.0	22.5	0.90	0.20	2	
ATCC 19115	Adhesion	Mango	AA	0.500000%	5.0	22.5	0.80	0.20	2	
ATCC 19115	Adhesion	Mango	SH	0.020000%	5.0	22.5	0.80	0.20	2	
Scott A	Adhesion	Stainless steel	SH	0.051200%	5.0	22.0	5.00	Unclear	3	[79]
Scott A	Adhesion	Stainless steel	SH	0.025600%	5.0	22.0	2.50	Unclear	3	
Scott A	Adhesion	Stainless steel	SH	0.012800%	5.0	22.0	1.40	Unclear	3	
Scott A	Adhesion	Stainless steel	CHD	0.025600%	5.0	22.0	3.10	Unclear	3	
Scott A	Adhesion	Stainless steel	CHD	0.012800%	5.0	22.0	2.20	Unclear	3	
Scott A	Adhesion	Stainless steel	BAC	0.025600%	5.0	22.0	3.70	Unclear	3	
Scott A	Adhesion	Stainless steel	BAC	0.012800%	5.0	22.0	2.40	Unclear	3	
Cocktail	Adhesion	Apple	EW	0.011000%	2.0	22.5	0.88	0.08	3	[80]
Cocktail	Adhesion	Apple	EW	0.011000%	0.5	22.5	0.75	0.09	3	
Cocktail	Adhesion	Apple	EW	0.011000%	2.0	22.5	0.93	0.09	3	
Cocktail	Adhesion	Apple	EW	0.011000%	0.5	22.5	0.70	0.10	3	
Cocktail	Adhesion	Apple	EW	0.011000%	0.5	22.5	0.70	0.00	3	[14]
Cocktail	Adhesion	Apple	EW	0.011000%	5.0	22.5	1.20	0.00	3	
Cocktail	Adhesion	Apple	CH	0.010000%	2.0	22.5	1.20	0.10	3	
Cocktail	Adhesion	Apple	PAA	0.008000%	2.0	22.5	1.80	0.10	3	
Cocktail	Adhesion	Apple	CH	0.010000%	0.5	22.5	0.70	0.10	3	

Cocktail	Adhesion	Apple	PAA	0.008000%	0.5	22.5	1.20	0.10	3	
Cocktail	Adhesion	Apple	CH	0.010000%	5.0	22.5	1.20	0.10	3	
Cocktail	Adhesion	Apple	PAA	0.008000%	5.0	22.5	1.80	0.10	3	
Cocktail	Adhesion	Apple	EW	0.011000%	2.0	22.5	1.10	0.20	3	
Cocktail	Adhesion	Apple	EW	0.011000%	2.0	22.5	1.30	0.20	3	
Cocktail	Adhesion	Apple	CH	0.011300%	2.0	22.5	1.15	0.01	3	[80]
Cocktail	Adhesion	Apple	CH	0.011300%	0.5	22.5	0.90	0.04	3	
Cocktail	Adhesion	Apple	CH	0.011300%	0.5	22.5	0.88	0.07	3	
Cocktail	Adhesion	Apple	CH	0.011300%	2.0	22.5	1.05	0.10	3	
Cocktail	Adhesion	Apple	SAS	1.000000%	2.0	22.0	1.33	0.02	3	[42]
Cocktail	Adhesion	Apple	SAS	1.000000%	5.0	22.0	1.33	0.02	3	
Cocktail	Adhesion	Apple	SAS	1.500000%	0.5	22.0	1.31	0.02	3	
Cocktail	Adhesion	Apple	SAS	1.500000%	5.0	22.0	1.94	0.02	3	
Cocktail	Adhesion	Apple	SAS	3.000000%	2.0	22.0	3.03	0.03	3	
Cocktail	Adhesion	Apple	SAS	2.000000%	5.0	22.0	2.41	0.04	3	
Cocktail	Adhesion	Apple	SAS	1.500000%	2.0	22.0	1.91	0.05	3	
Cocktail	Adhesion	Apple	SAS	3.000000%	5.0	22.0	3.16	0.05	3	
Cocktail	Adhesion	Apple	SAS	1.000000%	0.5	22.0	0.91	0.07	3	
Cocktail	Adhesion	Apple	SAS	2.000000%	2.0	22.0	2.28	0.09	3	
Cocktail	Adhesion	Apple	SAS	3.000000%	0.5	22.0	3.05	0.09	3	
Cocktail	Adhesion	Apple	SAS	2.000000%	0.5	22.0	2.09	0.20	3	
Cocktail	Adhesion	Cantaloupe	EW	0.010000%	5.0	22.5	2.10	0.00	6	[81]
Cocktail	Adhesion	Cantaloupe	PAA	0.010000%	5.0	22.5	4.50	0.10	2	
Cocktail	Adhesion	Cantaloupe	PAA	0.004500%	5.0	22.5	3.00	0.20	2	
Cocktail	Adhesion	Cantaloupe	EW	0.010000%	5.0	22.5	1.90	0.20	6	
Cocktail	Adhesion	Cantaloupe	LA	2.000000%	5.0	22.5	2.50	0.70	2	
Cocktail	Adhesion	Cantaloupe	PAA	0.008500%	5.0	22.5	3.00	1.20	2	
Cocktail	Adhesion	Lettuce	LA	2.000000%	5.0	22.5	1.70	0.10	2	
Cocktail	Adhesion	Lettuce	EW	0.010000%	5.0	22.5	1.70	0.10	6	
Cocktail	Adhesion	Lettuce	EW	0.010000%	5.0	22.5	2.00	0.10	6	
Cocktail	Adhesion	Lettuce	PAA	0.008500%	5.0	22.5	2.00	0.20	2	
Cocktail	Adhesion	Lettuce	PAA	0.004500%	5.0	22.5	2.00	0.30	2	

Cocktail	Adhesion	Lettuce	PAA	0.010000%	5.0	22.5	2.40	0.50	2	
Cocktail	Adhesion	Lettuce	SH	0.012000%	2.0	22.5	1.10	0.37	11	[82]
Cocktail	Adhesion	Apple	CO	0.200000%	3.0	23.0	1.78	0.00	2	[83]
Cocktail	Adhesion	Apple	FA	0.500000%	3.0	23.0	2.18	0.10	2	
Cocktail	Adhesion	Tomato	FA	0.500000%	3.0	23.0	2.18	0.04	2	
Cocktail	Adhesion	Strawberry	EW	0.004700%	5.0	22.0	1.65	0.47	4	[84]
Cocktail	Adhesion	Alfalfa sprout	OW	0.000500%	2.0	4.0	0.78	Unclear	3	[85]
Cocktail	Adhesion	Alfalfa sprout	OW	0.000900%	2.0	4.0	0.81	Unclear	3	
Cocktail	Adhesion	Alfalfa sprout	OW	0.002330%	2.0	4.0	0.91	Unclear	3	
CRA359	Adhesion	Stainless steel	SDS	0.200000%	20.0	22.5	2.06	Unclear	3	[86]
Unclear	Adhesion	Lotus	SH	0.020000%	10.0	22.5	1.15	Unclear	3	[87]
Cocktail	Adhesion	Alfalfa seed	HP	8.000000%	10.0	22.5	4.40	0.02	2	[88]
Cocktail	Adhesion	Alfalfa seed	CH	0.200000%	20.0	22.5	3.83	0.46	2	
Cocktail	Adhesion	Radish seed	HP	8.000000%	10.0	22.5	1.50	0.38	2	
Cocktail	Adhesion	Radish seed	CH	0.200000%	20.0	22.5	0.51	0.50	2	
Cocktail	Adhesion	Pleurotus eryngii	AA	1.000000%	10.0	25.0	0.67	Unclear	2	[89]
Cocktail	Adhesion	Pleurotus eryngii	AA	3.000000%	30.0	25.0	2.00	Unclear	2	
Cocktail	Adhesion	Pleurotus eryngii	LA	1.000000%	10.0	25.0	0.44	Unclear	2	
Cocktail	Adhesion	Pleurotus eryngii	LA	3.000000%	30.0	25.0	1.17	Unclear	2	
Cocktail	Adhesion	Pleurotus eryngii	MA	1.000000%	10.0	25.0	1.87	Unclear	2	
Cocktail	Adhesion	Pleurotus eryngii	MA	1.000000%	30.0	25.0	2.43	Unclear	2	
Cocktail	Adhesion	Pleurotus eryngii	MA	2.000000%	30.0	25.0	3.01	Unclear	2	
Cocktail	Adhesion	Pleurotus eryngii	MA	3.000000%	30.0	25.0	3.04	Unclear	2	
Cocktail	Adhesion	Enoki mushroom	AA	1.000000%	5.0	22.0	0.59	Unclear	2	[90]
Cocktail	Adhesion	Enoki mushroom	CA	1.000000%	5.0	22.0	0.51	Unclear	2	
Cocktail	Adhesion	Enoki mushroom	LA	1.000000%	5.0	22.0	0.71	Unclear	2	
Cocktail	Biofilm	Stainless steel	SD	0.040000%	0.5	22.5	3.10	Unclear	2	[91]
EGDe	Biofilm	Stainless steel	EW	0.004000%	3.0	25.0	1.16	0.10	3	[92]
EGDe	Biofilm	Stainless steel	EW	0.004000%	3.0	25.0	2.31	0.22	3	
Cocktail	Biofilm	Stainless steel	EW	0.004712%	0.5	24.0	4.33	0.02	5	[93]
Cocktail	Biofilm	Stainless steel	EW	0.008468%	2.0	24.0	5.13	0.03	5	
Cocktail	Biofilm	Stainless steel	EW	0.008468%	1.0	24.0	4.81	0.04	5	

Cocktail	Biofilm	Stainless steel	EW	0.004712%	2.0	24.0	5.21	0.07	5	
Cocktail	Biofilm	Stainless steel	EW	0.008468%	0.5	24.0	4.56	0.15	5	
Cocktail	Biofilm	Stainless steel	EW	0.004712%	1.0	24.0	4.65	0.22	5	
Cocktail	Biofilm	Stainless steel	BAC	0.002000%	0.5	22.0	0.42	0.03	3	[94]
Cocktail	Biofilm	Stainless steel	I	0.005000%	0.3	22.0	0.97	0.04	3	
Cocktail	Biofilm	Stainless steel	BAC	0.010000%	0.3	22.0	0.59	0.04	3	
Cocktail	Biofilm	Stainless steel	BAC	0.002000%	0.1	22.0	0.12	0.05	3	
Cocktail	Biofilm	Stainless steel	SH	0.010000%	0.5	22.0	1.34	0.05	3	
Cocktail	Biofilm	Stainless steel	I	0.005000%	0.1	22.0	0.57	0.06	3	
Cocktail	Biofilm	Stainless steel	SH	0.005000%	0.3	22.0	0.82	0.07	3	
Cocktail	Biofilm	Stainless steel	BAC	0.005000%	0.1	22.0	0.19	0.07	3	
Cocktail	Biofilm	Stainless steel	SH	0.010000%	0.3	22.0	1.21	0.07	3	
Cocktail	Biofilm	Stainless steel	BAC	0.010000%	0.1	22.0	0.28	0.07	3	
Cocktail	Biofilm	Stainless steel	SH	0.002000%	0.3	22.0	0.34	0.08	3	
Cocktail	Biofilm	Stainless steel	SH	0.002000%	0.5	22.0	0.44	0.08	3	
Cocktail	Biofilm	Stainless steel	BAC	0.002000%	0.3	22.0	0.24	0.08	3	
Cocktail	Biofilm	Stainless steel	SH	0.005000%	0.1	22.0	0.39	0.08	3	
Cocktail	Biofilm	Stainless steel	I	0.010000%	0.5	22.0	1.40	0.10	3	
Cocktail	Biofilm	Stainless steel	SH	0.002000%	0.1	22.0	0.30	0.11	3	
Cocktail	Biofilm	Stainless steel	I	0.002000%	0.5	22.0	0.64	0.11	3	
Cocktail	Biofilm	Stainless steel	BAC	0.010000%	0.5	22.0	0.72	0.11	3	
Cocktail	Biofilm	Stainless steel	BAC	0.005000%	0.3	22.0	0.39	0.12	3	
Cocktail	Biofilm	Stainless steel	I	0.002000%	0.1	22.0	0.25	0.13	3	
Cocktail	Biofilm	Stainless steel	HP	0.500000%	0.1	22.0	0.30	0.15	3	
Cocktail	Biofilm	Stainless steel	HP	1.000000%	0.1	22.0	0.30	0.15	3	
Cocktail	Biofilm	Stainless steel	HP	2.000000%	0.5	22.0	1.17	0.15	3	
Cocktail	Biofilm	Stainless steel	BAC	0.005000%	0.5	22.0	0.58	0.16	3	
Cocktail	Biofilm	Stainless steel	SH	0.005000%	0.5	22.0	1.06	0.18	3	
Cocktail	Biofilm	Stainless steel	SH	0.010000%	0.1	22.0	0.61	0.19	3	
Cocktail	Biofilm	Stainless steel	HP	2.000000%	0.3	22.0	1.01	0.19	3	
Cocktail	Biofilm	Stainless steel	HP	0.500000%	0.3	22.0	0.51	0.20	3	
Cocktail	Biofilm	Stainless steel	HP	1.000000%	0.3	22.0	0.51	0.20	3	

Cocktail	Biofilm	Stainless steel	I	0.002000%	0.3	22.0	0.35	0.22	3	
Cocktail	Biofilm	Stainless steel	I	0.005000%	0.5	22.0	1.20	0.22	3	
Cocktail	Biofilm	Stainless steel	HP	0.500000%	0.5	22.0	0.71	0.28	3	
Cocktail	Biofilm	Stainless steel	HP	1.000000%	0.5	22.0	0.71	0.28	3	
Cocktail	Biofilm	Stainless steel	HP	2.000000%	0.1	22.0	0.80	0.28	3	
Cocktail	Biofilm	Stainless steel	I	0.010000%	0.1	22.0	0.81	0.34	3	
Cocktail	Biofilm	Stainless steel	I	0.010000%	0.3	22.0	1.13	0.42	3	
ATCC 7677	Biofilm	Stainless steel	SSP	0.250000%	1.0	22.5	2.58	Unclear	3	[95]
ATCC 7677	Biofilm	Stainless steel	SSP	0.500000%	1.0	22.5	4.90	Unclear	3	
ATCC 7677	Biofilm	Stainless steel	SSP	0.500000%	5.0	22.5	5.35	Unclear	3	
ATCC 7677	Biofilm	Stainless steel	SSP	0.500000%	10.0	22.5	5.50	Unclear	3	
ATCC 7677	Biofilm	Stainless steel	SSP	0.250000%	10.0	22.5	4.10	Unclear	3	
ATCC 7677	Biofilm	Stainless steel	SSP	0.500000%	10.0	22.5	4.70	Unclear	3	
ATCC 7644	Biofilm	Stainless steel	SH	1.500000%	10.0	25.0	5.30	0.00	3	[96]
ATCC 7644	Biofilm	Stainless steel	IO	0.200000%	10.0	25.0	4.30	0.00	3	
ATCC 7644	Biofilm	Stainless steel	PAA	0.500000%	10.0	25.0	5.20	0.10	3	
Cocktail	Biofilm	Stainless steel	LA	3.000000%	10.0	22.5	4.20	0.00	3	[97]
Cocktail	Biofilm	Stainless steel	HP	2.000000%	10.0	22.5	0.30	0.00	3	
Cocktail	Biofilm	Stainless steel	LVA	3.000000%	10.0	22.5	0.30	0.20	3	
Cocktail	Biofilm	Stainless steel	SH	0.010000%	10.0	22.5	0.70	0.30	3	
Cocktail	Biofilm	Stainless steel	SDS	2.000000%	10.0	22.5	1.20	0.50	3	
Scott A	Biofilm	Stainless steel	SHD	0.004000%	60.0	16.0	0.12	0.01	4	[98]
Scott A	Biofilm	Stainless steel	LA	0.009000%	180.0	16.0	0.71	0.02	4	
Scott A	Biofilm	Stainless steel	SHD	0.004000%	180.0	16.0	0.24	0.03	4	
Scott A	Biofilm	Stainless steel	LA	0.009000%	60.0	16.0	0.47	0.05	4	
Scott A	Biofilm	Stainless steel	HA	0.003650%	60.0	16.0	0.16	0.10	4	
Scott A	Biofilm	Stainless steel	HA	0.003650%	180.0	16.0	0.54	0.18	4	
Cocktail	Biofilm	Stainless steel	SH	0.003600%	3.0	22.0	3.00	0.10	4	[16]
Cocktail	Biofilm	Stainless steel	SH	0.001400%	3.0	22.0	1.80	0.20	4	
Cocktail	Biofilm	Stainless steel	SH	0.007200%	3.0	22.0	4.10	0.20	4	
Cocktail	Biofilm	Stainless steel	SH	0.014400%	3.0	22.0	4.80	0.20	4	
Cocktail	Biofilm	Stainless steel	SH	0.014400%	1.0	22.0	3.60	0.30	4	

Cocktail	Biofilm	Stainless steel	SH	0.003600%	1.0	22.0	1.80	0.40	4	
Cocktail	Biofilm	Stainless steel	SH	0.007200%	1.0	22.0	2.90	0.40	4	
Cocktail	Biofilm	Stainless steel	SH	0.001400%	1.0	22.0	1.00	0.90	4	
Cocktail	Biofilm	Polyester	CDS	0.000250%	1.0	22.0	1.10	Unclear	3	[99]
Cocktail	Biofilm	Polyester	PAA	0.016000%	1.0	22.0	4.10	Unclear	3	
Cocktail	Biofilm	Polyester	PAA	0.020000%	5.0	22.0	4.30	Unclear	3	
Cocktail	Biofilm	Polyethylene	CDS	0.000250%	1.0	22.0	0.60	Unclear	3	
Cocktail	Biofilm	Polyethylene	PAA	0.016000%	1.0	22.0	3.50	Unclear	3	
Cocktail	Biofilm	Polyethylene	PAA	0.020000%	5.0	22.0	4.00	Unclear	3	
Cocktail	Biofilm	Polyvinyl chloride	CDS	0.000250%	1.0	22.0	0.90	Unclear	3	
Cocktail	Biofilm	Polyvinyl chloride	PAA	0.016000%	1.0	22.0	3.80	Unclear	3	
Cocktail	Biofilm	Polyvinyl chloride	PAA	0.020000%	5.0	22.0	4.40	Unclear	3	
Cocktail	Biofilm	Rubber	CDS	0.000250%	1.0	22.0	0.90	Unclear	3	
Cocktail	Biofilm	Rubber	PAA	0.016000%	1.0	22.0	3.70	Unclear	3	
Cocktail	Biofilm	Rubber	PAA	0.020000%	5.0	22.0	4.40	Unclear	3	
Cocktail	Biofilm	Stainless steel	CDS	0.000250%	1.0	22.0	1.10	Unclear	3	
Cocktail	Biofilm	Stainless steel	PAA	0.016000%	1.0	22.0	4.30	Unclear	3	
Cocktail	Biofilm	Stainless steel	PAA	0.020000%	5.0	22.0	4.50	Unclear	3	
ATCC 19113	Biofilm	Egg shell	SH	0.015000%	1.0	22.5	1.14	0.04	3	[45]
ATCC 19113	Biofilm	Egg shell	SH	0.020000%	1.0	22.5	1.26	0.17	3	
ATCC 19113	Biofilm	Egg shell	SH	0.010000%	1.0	22.5	1.07	0.22	3	
ATCC 19113	Biofilm	Egg shell	SH	0.005000%	1.0	22.5	0.24	0.31	3	
ATCC 19113	Biofilm	Stainless steel	SH	0.010000%	1.0	22.5	0.83	0.03	3	
ATCC 19113	Biofilm	Stainless steel	SH	0.015000%	1.0	22.5	1.45	0.04	3	
ATCC 19113	Biofilm	Stainless steel	SH	0.020000%	1.0	22.5	1.55	0.05	3	
ATCC 19113	Biofilm	Stainless steel	SH	0.005000%	1.0	22.5	0.34	0.19	3	
Dairy isolated	Biofilm	96-well polystyrene	CDS	0.000250%	1.0	22.0	0.52	0.01	3	[44]
Dairy isolated	Biofilm	96-well polystyrene	PAA	0.008000%	1.0	22.0	1.66	0.01	3	
Dairy isolated	Biofilm	96-well polystyrene	CDS	0.000250%	1.0	22.0	0.87	0.02	3	
Dairy isolated	Biofilm	96-well polystyrene	PAA	0.016000%	1.0	22.0	4.34	0.02	3	
Dairy isolated	Biofilm	96-well polystyrene	OW	0.000200%	1.0	22.0	0.16	0.02	3	
Dairy isolated	Biofilm	96-well polystyrene	OW	0.000200%	1.0	22.0	0.12	0.03	3	

Dairy isolated	Biofilm	96-well polystyrene	OW	0.000400%	1.0	22.0	0.29	0.03	3
Dairy isolated	Biofilm	96-well polystyrene	CDS	0.000250%	1.0	22.0	0.63	0.04	3
Dairy isolated	Biofilm	96-well polystyrene	OW	0.000400%	1.0	22.0	0.70	0.04	3
Dairy isolated	Biofilm	96-well polystyrene	OW	0.000200%	1.0	22.0	0.11	0.04	3
Dairy isolated	Biofilm	96-well polystyrene	OW	0.000400%	1.0	22.0	0.36	0.05	3
Dairy isolated	Biofilm	96-well polystyrene	OW	0.000400%	1.0	22.0	0.31	0.05	3
Dairy isolated	Biofilm	96-well polystyrene	OW	0.000200%	1.0	22.0	0.26	0.05	3
Dairy isolated	Biofilm	96-well polystyrene	PAA	0.008000%	1.0	22.0	3.29	0.05	3
Dairy isolated	Biofilm	96-well polystyrene	OW	0.000400%	1.0	22.0	0.24	0.05	3
Dairy isolated	Biofilm	96-well polystyrene	PAA	0.008000%	1.0	22.0	2.13	0.05	3
Dairy isolated	Biofilm	96-well polystyrene	PAA	0.020000%	1.0	22.0	3.50	0.05	3
Dairy isolated	Biofilm	96-well polystyrene	CDS	0.000250%	1.0	22.0	0.50	0.05	3
Dairy isolated	Biofilm	96-well polystyrene	CDS	0.000250%	1.0	22.0	2.31	0.06	3
Dairy isolated	Biofilm	96-well polystyrene	PAA	0.016000%	1.0	22.0	4.76	0.06	3
Dairy isolated	Biofilm	96-well polystyrene	CDS	0.000500%	1.0	22.0	2.23	0.06	3
Dairy isolated	Biofilm	96-well polystyrene	OW	0.000200%	1.0	22.0	0.20	0.06	3
Dairy isolated	Biofilm	96-well polystyrene	PAA	0.020000%	1.0	22.0	4.46	0.06	3
Dairy isolated	Biofilm	96-well polystyrene	CDS	0.000500%	1.0	22.0	1.48	0.07	3
Dairy isolated	Biofilm	96-well polystyrene	CDS	0.000500%	1.0	22.0	1.73	0.07	3
Dairy isolated	Biofilm	96-well polystyrene	CDS	0.000500%	1.0	22.0	1.31	0.07	3
Dairy isolated	Biofilm	96-well polystyrene	PAA	0.008000%	1.0	22.0	3.48	0.08	3
Dairy isolated	Biofilm	96-well polystyrene	PAA	0.016000%	1.0	22.0	3.36	0.08	3
Dairy isolated	Biofilm	96-well polystyrene	CDS	0.000500%	1.0	22.0	1.24	0.08	3
Dairy isolated	Biofilm	96-well polystyrene	PAA	0.016000%	1.0	22.0	3.17	0.08	3
Dairy isolated	Biofilm	96-well polystyrene	OW	0.000400%	1.0	22.0	2.27	0.09	3
Dairy isolated	Biofilm	96-well polystyrene	PAA	0.020000%	1.0	22.0	4.99	0.09	3
Dairy isolated	Biofilm	96-well polystyrene	CDS	0.000250%	1.0	22.0	0.90	0.09	3
Dairy isolated	Biofilm	96-well polystyrene	PAA	0.016000%	1.0	22.0	3.70	0.09	3
Dairy isolated	Biofilm	96-well polystyrene	PAA	0.020000%	1.0	22.0	3.31	0.09	3
Dairy isolated	Biofilm	96-well polystyrene	CDS	0.000500%	1.0	22.0	3.55	0.10	3
Dairy isolated	Biofilm	96-well polystyrene	OW	0.000200%	1.0	22.0	1.58	0.14	3
Dairy isolated	Biofilm	96-well polystyrene	PAA	0.016000%	1.0	22.0	3.62	0.15	3

Dairy isolated	Biofilm	96-well polystyrene	PAA	0.020000%	1.0	22.0	3.85	0.16	3	
Dairy isolated	Biofilm	96-well polystyrene	PAA	0.020000%	1.0	22.0	3.91	0.18	3	
Dairy isolated	Biofilm	96-well polystyrene	PAA	0.008000%	1.0	22.0	2.69	0.19	3	
Dairy isolated	Biofilm	96-well polystyrene	PAA	0.008000%	1.0	22.0	2.15	2.15	3	
Dairy isolated	Biofilm	96-well polystyrene	OW	0.000100%	1.0	22.0	0.90	Unclear	3	
Dairy isolated	Biofilm	96-well polystyrene	OW	0.000200%	1.0	22.0	3.40	Unclear	3	
Dairy isolated	Biofilm	96-well polystyrene	OW	0.000400%	1.0	22.0	4.10	Unclear	3	
Dairy isolated	Biofilm	96-well polystyrene	CDS	0.000250%	1.0	22.0	2.40	Unclear	3	
Dairy isolated	Biofilm	96-well polystyrene	CDS	0.000500%	1.0	22.0	3.20	Unclear	3	
Dairy isolated	Biofilm	96-well polystyrene	PAA	0.008000%	1.0	22.0	3.60	Unclear	3	
Dairy isolated	Biofilm	96-well polystyrene	PAA	0.016000%	1.0	22.0	4.80	Unclear	3	
Cocktail	Biofilm	Stainless steel	EA	20.000000%	5.0	22.5	0.00	Unclear	3	[100]
Cocktail	Biofilm	Stainless steel	EA	30.000000%	5.0	22.5	0.38	Unclear	3	
Cocktail	Biofilm	Stainless steel	EA	40.000000%	5.0	22.5	3.53	Unclear	3	
Cocktail	Biofilm	Stainless steel	EA	50.000000%	5.0	22.5	3.65	Unclear	3	
Cocktail	Biofilm	Stainless steel	EA	60.000000%	5.0	22.5	3.65	Unclear	3	
Cocktail	Biofilm	Stainless steel	EA	70.000000%	5.0	22.5	3.65	Unclear	3	
Cocktail	Biofilm	Stainless steel	EA	80.000000%	5.0	22.5	3.65	Unclear	3	
ATCC 5779	Biofilm	Polystyrene	PLA	3.000000%	5.0	21.0	4.06	Unclear	6	[101]
ATCC 5779	Biofilm	Polystyrene	PLA	1.000000%	5.0	21.0	3.94	Unclear	6	
ATCC 5779	Biofilm	Polystyrene	PLA	0.500000%	30.0	21.0	3.99	Unclear	6	
ATCC 5779	Biofilm	Polystyrene	PLA	0.500000%	60.0	21.0	5.10	Unclear	6	
ATCC 5779	Biofilm	Polystyrene	PLA	3.000000%	5.0	21.0	3.40	Unclear	6	
ATCC 5779	Biofilm	Polystyrene	PLA	1.000000%	5.0	21.0	0.92	Unclear	6	
ATCC 5779	Biofilm	Polystyrene	PLA	0.500000%	5.0	21.0	0.33	Unclear	6	
ATCC 5779	Biofilm	Polystyrene	PLA	0.250000%	5.0	21.0	0.20	Unclear	6	
ATCC 5779	Biofilm	Polystyrene	LA	3.000000%	5.0	21.0	0.88	Unclear	6	
ATCC 5779	Biofilm	Polystyrene	LA	1.000000%	5.0	21.0	0.28	Unclear	6	
ATCC 5779	Biofilm	Polystyrene	PLA	3.000000%	5.0	21.0	4.51	Unclear	6	
ATCC 5779	Biofilm	Polystyrene	PLA	1.000000%	5.0	21.0	4.07	Unclear	6	
ATCC 5779	Biofilm	Polystyrene	PLA	0.500000%	5.0	21.0	1.39	Unclear	6	
ATCC 5779	Biofilm	Polystyrene	PLA	0.250000%	5.0	21.0	0.24	Unclear	6	

ATCC 5779	Biofilm	Polystyrene	LA	3.000000%	5.0	21.0	2.07	Unclear	6	
ATCC 5779	Biofilm	Polystyrene	LA	1.000000%	5.0	21.0	0.36	Unclear	6	
ATCC 5779	Biofilm	Polystyrene	PLA	3.000000%	5.0	21.0	3.51	Unclear	6	
ATCC 5779	Biofilm	Polystyrene	PLA	1.000000%	5.0	21.0	1.09	Unclear	6	
ATCC 5779	Biofilm	Polystyrene	PLA	0.500000%	5.0	21.0	0.48	Unclear	6	
ATCC 5779	Biofilm	Polystyrene	PLA	0.250000%	5.0	21.0	0.35	Unclear	6	
ATCC 5779	Biofilm	Polystyrene	LA	3.000000%	5.0	21.0	0.94	Unclear	6	
ATCC 5779	Biofilm	Polystyrene	LA	1.000000%	5.0	21.0	0.36	Unclear	6	
Scott A	Biofilm	Shrimp carapace	SH	0.010000%	5.0	4.0	2.87	0.00	3	[43]
Scott A	Biofilm	Shrimp carapace	SH	0.010000%	10.0	4.0	2.87	0.00	3	
V7	Biofilm	Shrimp carapace	SH	0.010000%	5.0	4.0	2.35	0.00	3	
V7	Biofilm	Shrimp carapace	SH	0.010000%	10.0	4.0	2.35	0.00	3	
V7	Biofilm	Shrimp carapace	SH	0.010000%	10.0	25.0	2.56	0.00	3	
V7	Biofilm	Shrimp carapace	SH	0.010000%	5.0	25.0	2.56	0.04	3	
V7	Biofilm	Shrimp carapace	SH	0.010000%	20.0	25.0	3.35	0.11	3	
Scott A	Biofilm	Shrimp carapace	SH	0.010000%	3.0	25.0	2.13	0.18	3	
Scott A	Biofilm	Shrimp carapace	SH	0.010000%	20.0	4.0	3.00	0.22	3	
Scott A	Biofilm	Shrimp carapace	SH	0.010000%	0.5	25.0	1.41	0.23	3	
V7	Biofilm	Shrimp carapace	SH	0.010000%	20.0	4.0	2.54	0.33	3	
V7	Biofilm	Shrimp carapace	SH	0.010000%	3.0	25.0	1.92	0.36	3	
Scott A	Biofilm	Shrimp carapace	SH	0.010000%	5.0	25.0	1.79	0.38	3	
V7	Biofilm	Shrimp carapace	SH	0.010000%	0.5	25.0	1.04	0.40	3	
Scott A	Biofilm	Shrimp carapace	SH	0.010000%	10.0	25.0	2.81	0.42	3	
V7	Biofilm	Shrimp carapace	SH	0.010000%	0.5	4.0	0.64	0.47	3	
Scott A	Biofilm	Shrimp carapace	SH	0.010000%	0.5	4.0	0.54	0.48	3	
V7	Biofilm	Shrimp carapace	SH	0.010000%	3.0	4.0	1.15	0.48	3	
Scott A	Biofilm	Shrimp carapace	SH	0.010000%	20.0	25.0	3.61	0.50	3	
Scott A	Biofilm	Shrimp carapace	SH	0.010000%	3.0	4.0	1.78	0.84	3	
Fish isolated	Biofilm	Stainless steel	BAC	0.010000%	15.0	22.5	5.42	0.27	6	[102]
Cocktail	Biofilm	Polystyrene	PAA	0.200000%	5.0	22.5	2.80	Unclear	2	[103]
Cocktail	Biofilm	Stainless steel	PAA	0.200000%	5.0	22.5	3.50	Unclear	2	
10403S	Biofilm	Stainless steel	CH	0.010000%	5.0	20.0	5.79	Unclear	3	[104]

10403S	Biofilm	Stainless steel	CH	0.015000%	1.0	20.0	6.27	Unclear	3	
Scott A	Biofilm	Stainless steel	HP	5.000000%	10.0	20.0	4.14	Unclear	3	
Scott A	Biofilm	Stainless steel	HP	5.000000%	15.0	20.0	5.58	Unclear	3	
Scott A	Biofilm	Stainless steel	HP	6.000000%	10.0	20.0	5.06	Unclear	3	
Scott A	Biofilm	Stainless steel	HP	6.000000%	15.0	20.0	5.58	Unclear	3	
Fish isolated	Biofilm	Aluminium foil	SHD	0.500000%	1.0	22.5	0.52	Unclear	3	[15]
Fish isolated	Biofilm	Aluminium foil	SH	0.500000%	1.0	22.5	3.84	Unclear	3	
Fish isolated	Biofilm	Aluminium foil	SHD	0.500000%	5.0	22.5	0.98	Unclear	3	
Fish isolated	Biofilm	Aluminium foil	SH	0.500000%	5.0	22.5	4.63	Unclear	3	
Fish isolated	Biofilm	Polypropylene	SHD	0.500000%	1.0	22.5	1.20	Unclear	3	
Fish isolated	Biofilm	Polypropylene	SH	0.500000%	1.0	22.5	2.74	Unclear	3	
Fish isolated	Biofilm	Polypropylene	SHD	0.500000%	5.0	22.5	1.81	Unclear	3	
Fish isolated	Biofilm	Polypropylene	SH	0.500000%	5.0	22.5	3.26	Unclear	3	
Fish isolated	Biofilm	Rubber	SHD	0.500000%	1.0	22.5	0.66	Unclear	3	
Fish isolated	Biofilm	Rubber	SH	0.500000%	1.0	22.5	1.79	Unclear	3	
Fish isolated	Biofilm	Rubber	SHD	0.500000%	5.0	22.5	1.63	Unclear	3	
Fish isolated	Biofilm	Rubber	SH	0.500000%	5.0	22.5	2.21	Unclear	3	
Fish isolated	Biofilm	Stainless steel	SHD	0.500000%	1.0	22.5	1.00	Unclear	3	
Fish isolated	Biofilm	Stainless steel	SH	0.500000%	1.0	22.5	1.97	Unclear	3	
Fish isolated	Biofilm	Stainless steel	SHD	0.500000%	5.0	22.5	1.00	Unclear	3	
Fish isolated	Biofilm	Stainless steel	SH	0.500000%	5.0	22.5	3.55	Unclear	3	
Cocktail	Biofilm	96-well polystyrene	TDS	0.100000%	1.0	22.5	1.30	0.04	3	[105]
Cocktail	Biofilm	96-well polystyrene	TDS	0.050000%	1.0	22.5	0.89	0.07	3	
Cocktail	Biofilm	96-well polystyrene	TDS	0.010000%	1.0	22.5	0.00	0.09	3	
Meat isolated	Biofilm	Stainless steel	PAA	0.015000%	2.0	22.5	3.10	0.00	4	[106]
Meat isolated	Biofilm	Stainless steel	SH	0.020000%	5.0	22.5	3.00	0.10	4	
Meat isolated	Biofilm	Stainless steel	SH	0.020000%	0.5	22.5	2.10	0.10	4	
Meat isolated	Biofilm	Stainless steel	SH	0.020000%	2.0	22.5	2.60	0.20	4	
Meat isolated	Biofilm	Stainless steel	SH	0.020000%	5.0	22.5	3.00	0.20	4	
Meat isolated	Biofilm	Stainless steel	PAA	0.015000%	0.5	22.5	3.20	0.30	4	
Meat isolated	Biofilm	Stainless steel	SH	0.020000%	2.0	22.5	2.20	0.30	4	
Meat isolated	Biofilm	Stainless steel	PAA	0.015000%	0.5	22.5	2.00	0.50	4	

Meat isolated	Biofilm	Stainless steel	SH	0.020000%	0.5	22.5	1.50	0.60	4	
Meat isolated	Biofilm	Stainless steel	SH	0.020000%	0.5	22.5	1.20	0.90	4	
Cocktail	Biofilm	Stainless steel	CDS	0.000700%	2.0	22.0	2.29	0.14	3	[107]
Cocktail	Biofilm	Stainless steel	SH	0.005000%	1.0	22.0	0.78	0.27	3	
Cocktail	Biofilm	Stainless steel	SH	0.005000%	5.0	22.0	1.60	0.39	3	
Cocktail	Biofilm	Stainless steel	SH	0.005000%	10.0	22.0	3.09	0.49	3	
Cocktail	Biofilm	Stainless steel	CDS	0.000700%	4.0	22.0	3.07	0.54	3	
Cocktail	Biofilm	Stainless steel	CDS	0.000700%	8.0	22.0	3.49	0.57	3	
Cocktail	Biofilm	Stainless steel	CDS	0.000700%	6.0	22.0	3.07	0.63	3	
Cocktail	Biofilm	Stainless steel	CDS	0.000700%	10.0	22.0	3.74	0.64	3	
EGDe	Planktonic	Deionized water	EW	0.000200%	5.0	25.0	1.44	0.03	3	[92]
EGDe	Planktonic	Deionized water	EW	0.000400%	1.0	25.0	2.18	0.13	3	
EGDe	Planktonic	Deionized water	EW	0.000400%	5.0	25.0	2.10	0.18	3	
EGDe	Planktonic	Deionized water	EW	0.000500%	3.0	25.0	1.36	0.24	3	
EGDe	Planktonic	Deionized water	EW	0.000400%	3.0	25.0	2.10	0.35	3	
EGDe	Planktonic	Deionized water	EW	0.000500%	1.0	25.0	1.45	0.36	3	
EGDe	Planktonic	Deionized water	EW	0.000700%	3.0	25.0	1.86	0.38	3	
EGDe	Planktonic	Deionized water	EW	0.000200%	3.0	25.0	0.81	0.41	3	
EGDe	Planktonic	Deionized water	EW	0.000500%	5.0	25.0	1.20	0.45	3	
EGDe	Planktonic	Deionized water	EW	0.000700%	5.0	25.0	2.67	0.59	3	
EGDe	Planktonic	Deionized water	EW	0.000700%	1.0	25.0	1.39	0.70	3	
EGDe	Planktonic	Deionized water	EW	0.000200%	1.0	25.0	0.86	0.90	3	
Scott A	Planktonic	Saline solution	PAA	0.000500%	0.5	20.0	0.06	Unclear	6	[108]
Scott A	Planktonic	Saline solution	PAA	0.000500%	1.0	20.0	0.10	Unclear	6	
Scott A	Planktonic	Saline solution	PAA	0.000500%	5.0	20.0	2.15	Unclear	6	
Scott A	Planktonic	Saline solution	PAA	0.000500%	0.5	4.0	0.03	Unclear	6	
Scott A	Planktonic	Saline solution	PAA	0.000500%	1.0	4.0	0.06	Unclear	6	
Scott A	Planktonic	Saline solution	PAA	0.000500%	5.0	4.0	0.28	Unclear	6	
Scott A	Planktonic	Saline solution	PAA	0.000500%	15.0	4.0	0.33	Unclear	6	
Scott A	Planktonic	Saline solution	PAA	0.000500%	30.0	4.0	0.32	Unclear	6	
Scott A	Planktonic	Saline solution	PAA	0.001000%	1.0	20.0	0.04	Unclear	6	
Scott A	Planktonic	Saline solution	PAA	0.001000%	5.0	20.0	2.90	Unclear	6	

Scott A	Planktonic	Saline solution	PAA	0.001000%	0.5	4.0	0.19	Unclear	6	
Scott A	Planktonic	Saline solution	PAA	0.001000%	1.0	4.0	0.10	Unclear	6	
Scott A	Planktonic	Saline solution	PAA	0.001000%	5.0	4.0	0.86	Unclear	6	
Scott A	Planktonic	Saline solution	PAA	0.001000%	15.0	4.0	3.56	Unclear	6	
Scott A	Planktonic	Saline solution	PAA	0.001000%	30.0	4.0	5.91	Unclear	6	
Scott A	Planktonic	Saline solution	PAA	0.002000%	0.5	20.0	1.44	Unclear	6	
Scott A	Planktonic	Saline solution	PAA	0.002000%	1.0	20.0	2.63	Unclear	6	
Scott A	Planktonic	Saline solution	PAA	0.002000%	5.0	20.0	4.20	Unclear	6	
Scott A	Planktonic	Saline solution	PAA	0.002000%	1.0	4.0	0.22	Unclear	6	
Scott A	Planktonic	Saline solution	PAA	0.002000%	5.0	4.0	3.76	Unclear	6	
Scott A	Planktonic	Saline solution	PAA	0.003000%	0.5	20.0	1.74	Unclear	6	
Scott A	Planktonic	Saline solution	PAA	0.003000%	1.0	20.0	2.21	Unclear	6	
Scott A	Planktonic	Saline solution	PAA	0.003000%	5.0	20.0	2.73	Unclear	6	
Scott A	Planktonic	Saline solution	PAA	0.003000%	0.5	4.0	0.09	Unclear	6	
Scott A	Planktonic	Saline solution	PAA	0.003000%	1.0	4.0	0.42	Unclear	6	
Scott A	Planktonic	Saline solution	PAA	0.004000%	0.5	20.0	1.36	Unclear	6	
Scott A	Planktonic	Saline solution	PAA	0.004000%	1.0	20.0	3.05	Unclear	6	
Scott A	Planktonic	Saline solution	PAA	0.004000%	0.5	4.0	0.76	Unclear	6	
Scott A	Planktonic	Saline solution	PAA	0.004000%	1.0	4.0	2.52	Unclear	6	
EGDe	Planktonic	Saline solution	PAA	0.000540%	5.0	20.0	2.00	Unclear	2	[109]
EGDe	Planktonic	Saline solution	PAA	0.000680%	5.0	20.0	3.00	Unclear	2	
EGDe	Planktonic	Saline solution	PAA	0.000910%	5.0	20.0	5.00	Unclear	2	
EGDe	Planktonic	Saline solution	BAC	0.001600%	5.0	20.0	2.00	Unclear	2	
EGDe	Planktonic	Saline solution	BAC	0.002000%	5.0	20.0	3.00	Unclear	2	
EGDe	Planktonic	Saline solution	BAC	0.002800%	5.0	20.0	5.00	Unclear	2	
EGDe	Planktonic	Saline solution	OPA	0.011000%	5.0	20.0	2.00	Unclear	2	
EGDe	Planktonic	Saline solution	OPA	0.024500%	5.0	20.0	3.00	Unclear	2	
EGDe	Planktonic	Saline solution	OPA	0.067000%	5.0	20.0	5.00	Unclear	2	
Scott A	Planktonic	TSB	SB	0.050000%	30.0	21.0	0.50	0.21	8	[54]
Scott A	Planktonic	TSB	SB	0.100000%	30.0	21.0	0.51	0.25	8	
Scott A	Planktonic	TSB	SB	0.050000%	30.0	21.0	2.44	0.60	8	
Scott A	Planktonic	TSB	SB	0.100000%	30.0	21.0	4.57	0.71	8	

Scott A	Planktonic	TSB	SB	0.050000%	30.0	21.0	4.24	0.84	8	
Scott A	Planktonic	TSB	SB	0.100000%	30.0	21.0	3.17	1.25	8	
Bug600	Planktonic	Deionized water	EA	20.000000%	60.0	22.5	5.30	0.00	2	[110]
Bug600	Planktonic	Deionized water	SH	0.100000%	60.0	22.5	5.60	0.20	2	
Bug600	Planktonic	Deionized water	HP	0.120000%	120.0	22.5	4.00	0.20	2	
Scott A	Planktonic	Deionized water	SH	0.100000%	60.0	22.5	6.10	0.30	2	
Scott A	Planktonic	Deionized water	EA	20.000000%	60.0	22.5	5.90	0.30	2	
Scott A	Planktonic	Deionized water	HP	0.120000%	120.0	22.5	2.90	0.40	2	
Bug600	Planktonic	TSBYE	EA	20.000000%	60.0	22.5	5.30	0.00	2	
Bug600	Planktonic	TSBYE	HP	0.120000%	120.0	22.5	3.60	0.20	2	
Scott A	Planktonic	TSBYE	SH	0.100000%	60.0	22.5	5.20	0.20	2	
Bug600	Planktonic	TSBYE	SH	0.100000%	60.0	22.5	4.90	0.30	2	
Scott A	Planktonic	TSBYE	HP	0.120000%	120.0	22.5	4.00	0.30	2	
Bug600	Planktonic	TSBYE	IA	18.000000%	60.0	22.5	4.90	0.30	2	
Scott A	Planktonic	TSBYE	IA	18.000000%	60.0	22.5	5.40	0.60	2	
Scott A	Planktonic	TSBYE	EA	20.000000%	60.0	22.5	5.90	0.80	2	
Dairy isolated	Planktonic	Unclear	BAC	0.120000%	5.0	20.0	5.21	0.00	1	[111]
Dairy isolated	Planktonic	Unclear	BAC	0.250000%	5.0	20.0	5.89	0.00	1	
Dairy isolated	Planktonic	Unclear	BAC	0.500000%	5.0	20.0	6.43	0.00	1	
Dairy isolated	Planktonic	Unclear	BAC	1.000000%	5.0	20.0	6.98	0.00	1	
Dairy isolated	Planktonic	Unclear	BAC	0.120000%	10.0	20.0	6.54	0.00	1	
Dairy isolated	Planktonic	Unclear	BAC	0.250000%	10.0	20.0	6.78	0.00	1	
Dairy isolated	Planktonic	Unclear	BAC	0.500000%	10.0	20.0	7.23	0.00	1	
Dairy isolated	Planktonic	Unclear	BAC	1.000000%	10.0	20.0	7.76	0.00	1	
Dairy isolated	Planktonic	Unclear	BAC	0.120000%	5.0	20.0	2.13	0.00	1	
Dairy isolated	Planktonic	Unclear	BAC	0.250000%	5.0	20.0	2.63	0.00	1	
Dairy isolated	Planktonic	Unclear	BAC	0.500000%	5.0	20.0	3.22	0.00	1	
Dairy isolated	Planktonic	Unclear	BAC	1.000000%	5.0	20.0	3.67	0.00	1	
Dairy isolated	Planktonic	Unclear	BAC	0.120000%	10.0	20.0	2.87	0.00	1	
Dairy isolated	Planktonic	Unclear	BAC	0.250000%	10.0	20.0	3.28	0.00	1	
Dairy isolated	Planktonic	Unclear	BAC	0.500000%	10.0	20.0	3.65	0.00	1	
Dairy isolated	Planktonic	Unclear	BAC	1.000000%	10.0	20.0	3.85	0.00	1	

ATCC 19115	Planktonic	BPW	EW	0.002900%	3.0	22.5	9.19	0.00	3	[112]
ATCC 19115	Planktonic	BPW	EW	0.002900%	5.0	22.5	9.23	0.00	3	
ATCC 19115	Planktonic	BPW	EW	0.000700%	1.0	22.5	1.65	0.03	3	
ATCC 19115	Planktonic	BPW	EW	0.002900%	1.0	22.5	7.26	0.03	3	
ATCC 19115	Planktonic	BPW	EW	0.000800%	1.0	22.5	1.69	0.04	3	
ATCC 19115	Planktonic	BPW	EW	0.001500%	1.0	22.5	4.95	0.05	3	
ATCC 19115	Planktonic	BPW	EW	0.002900%	1.0	40.0	7.51	0.05	3	
ATCC 19115	Planktonic	BPW	EW	0.002700%	1.0	22.5	7.12	0.06	3	
ATCC 19115	Planktonic	BPW	EW	0.003000%	1.0	22.5	6.45	0.07	3	
ATCC 19115	Planktonic	BPW	EW	0.001400%	1.0	22.5	4.32	0.08	3	
ATCC 19115	Planktonic	BPW	EW	0.001700%	1.0	22.5	5.43	0.08	3	
ATCC 19115	Planktonic	BPW	EW	0.002900%	1.0	22.5	7.34	0.08	3	
ATCC 19115	Planktonic	BPW	EW	0.002900%	1.0	18.0	7.34	0.08	3	
ATCC 19115	Planktonic	BPW	EW	0.003000%	1.0	22.5	7.04	0.09	3	
ATCC 19115	Planktonic	BPW	EW	0.000900%	1.0	22.5	1.71	0.09	3	
ATCC 19115	Planktonic	BPW	EW	0.002300%	1.0	22.5	6.67	0.09	3	
ATCC 19115	Planktonic	BPW	EW	0.002700%	1.0	40.0	7.31	0.09	3	
Cow isolated	Planktonic	Deionized water	PA	0.000256%	5.0	2.5	7.83	0.65	4	[113]
BCRC 14846	Planktonic	Deionized water	CDS	0.000013%	25.0	25.0	3.08	Unclear	2	[40]
BCRC 14846	Planktonic	Deionized water	CDS	0.000013%	5.0	25.0	1.45	Unclear	2	
BCRC 14846	Planktonic	Deionized water	CDS	0.000013%	5.0	40.0	4.62	Unclear	2	
BCRC 14846	Planktonic	Deionized water	CDS	0.000013%	15.0	25.0	2.62	Unclear	2	
ATCC 5779	Planktonic	Deionized water	PLA	0.250000%	60.0	21.0	0.41	0.01	6	[101]
ATCC 5779	Planktonic	Deionized water	PLA	0.500000%	1.0	21.0	0.05	0.02	6	
ATCC 5779	Planktonic	Deionized water	PLA	0.500000%	5.0	21.0	1.41	0.02	6	
ATCC 5779	Planktonic	Deionized water	PLA	0.500000%	10.0	21.0	2.16	0.02	6	
ATCC 5779	Planktonic	Deionized water	LA	3.000000%	1.0	21.0	0.81	0.02	6	
ATCC 5779	Planktonic	Deionized water	LA	1.000000%	2.0	21.0	0.10	0.02	6	
ATCC 5779	Planktonic	Deionized water	LA	1.000000%	5.0	21.0	0.28	0.02	6	
ATCC 5779	Planktonic	Deionized water	LA	1.000000%	10.0	21.0	0.46	0.02	6	
ATCC 5779	Planktonic	Deionized water	LA	1.000000%	30.0	21.0	0.81	0.02	6	
ATCC 5779	Planktonic	Deionized water	LA	3.000000%	5.0	21.0	1.70	0.03	6	

ATCC 5779	Planktonic	Deionized water	PLA	0.250000%	2.0	21.0	0.07	0.04	6	
ATCC 5779	Planktonic	Deionized water	PLA	0.250000%	30.0	21.0	0.18	0.04	6	
ATCC 5779	Planktonic	Deionized water	PLA	0.250000%	10.0	21.0	0.11	0.05	6	
ATCC 5779	Planktonic	Deionized water	LA	1.000000%	20.0	21.0	0.74	0.05	6	
ATCC 5779	Planktonic	Deionized water	LA	3.000000%	2.0	21.0	1.05	0.06	6	
ATCC 5779	Planktonic	Deionized water	LA	1.000000%	60.0	21.0	1.29	0.06	6	
ATCC 5779	Planktonic	Deionized water	PLA	0.250000%	5.0	21.0	0.10	0.07	6	
ATCC 5779	Planktonic	Deionized water	PLA	0.500000%	2.0	21.0	0.54	0.08	6	
ATCC 5779	Planktonic	Deionized water	LA	1.000000%	1.0	21.0	0.17	0.08	6	
ATCC 5779	Planktonic	Deionized water	PLA	0.250000%	1.0	21.0	0.00	0.09	6	
ATCC 5779	Planktonic	Deionized water	PLA	0.500000%	20.0	21.0	4.59	0.12	6	
ATCC 5779	Planktonic	Deionized water	PLA	0.250000%	20.0	21.0	0.10	0.16	6	
ATCC 5779	Planktonic	Deionized water	LA	3.000000%	10.0	21.0	2.18	0.18	6	
Unclear	Planktonic	TSBYE	BAC	0.005000%	20.0	22.0	4.20	0.00	3	[39]
Unclear	Planktonic	TSBYE	HP	1.500000%	20.0	22.0	4.70	0.00	3	
Unclear	Planktonic	TSBYE	BAC	0.005000%	20.0	22.0	3.50	0.00	3	
Unclear	Planktonic	TSBYE	HP	1.500000%	20.0	22.0	3.50	0.00	3	
Unclear	Planktonic	TSBYE	BAC	0.005000%	20.0	22.0	2.50	0.00	3	
Unclear	Planktonic	TSBYE	HP	1.500000%	20.0	22.0	3.50	0.00	3	
Unclear	Planktonic	TSBYE	BAC	0.005000%	20.0	22.0	2.90	0.00	3	
Unclear	Planktonic	TSBYE	HP	1.500000%	20.0	22.0	2.60	0.00	3	
Unclear	Planktonic	TSBYE	BAC	0.005000%	20.0	22.0	4.00	0.10	3	
Unclear	Planktonic	TSBYE	HP	1.500000%	20.0	22.0	4.00	0.10	3	
Unclear	Planktonic	TSBYE	HP	1.500000%	20.0	22.0	3.70	0.10	3	
Unclear	Planktonic	TSBYE	BAC	0.005000%	20.0	22.0	3.50	0.10	3	
Unclear	Planktonic	TSBYE	HP	1.500000%	20.0	22.0	3.60	0.10	3	
Unclear	Planktonic	TSBYE	BAC	0.005000%	20.0	22.0	3.70	0.10	3	
Unclear	Planktonic	TSBYE	HP	1.500000%	20.0	22.0	3.50	0.10	3	
Unclear	Planktonic	TSBYE	HP	1.500000%	20.0	22.0	2.80	0.10	3	
Unclear	Planktonic	TSBYE	HP	1.500000%	20.0	22.0	2.70	0.10	3	
Unclear	Planktonic	TSBYE	BAC	0.005000%	20.0	22.0	3.70	0.10	3	
Unclear	Planktonic	TSBYE	BAC	0.005000%	20.0	22.0	3.30	0.10	3	

Unclear	Planktonic	TSBYE	HP	1.500000%	20.0	22.0	2.80	0.10	3
Unclear	Planktonic	TSBYE	HP	1.500000%	20.0	22.0	3.70	0.10	3
Unclear	Planktonic	TSBYE	BAC	0.005000%	20.0	22.0	5.30	0.10	3
Unclear	Planktonic	TSBYE	HP	1.500000%	20.0	22.0	2.80	0.10	3
Unclear	Planktonic	TSBYE	BAC	0.005000%	20.0	22.0	3.60	0.10	3
Unclear	Planktonic	TSBYE	HP	1.500000%	20.0	22.0	3.80	0.10	3
Unclear	Planktonic	TSBYE	HP	1.500000%	20.0	22.0	2.10	0.10	3
Unclear	Planktonic	TSBYE	HP	1.500000%	20.0	22.0	3.20	0.10	3
Unclear	Planktonic	TSBYE	BAC	0.005000%	20.0	22.0	3.30	0.10	3
Unclear	Planktonic	TSBYE	HP	1.500000%	20.0	22.0	3.30	0.10	3
Unclear	Planktonic	TSBYE	HP	1.500000%	20.0	22.0	3.60	0.10	3
Unclear	Planktonic	TSBYE	BAC	0.005000%	20.0	22.0	3.80	0.20	3
Unclear	Planktonic	TSBYE	BAC	0.005000%	20.0	22.0	2.90	0.20	3
Unclear	Planktonic	TSBYE	HP	1.500000%	20.0	22.0	3.80	0.20	3
Unclear	Planktonic	TSBYE	BAC	0.005000%	20.0	22.0	4.00	0.20	3
Unclear	Planktonic	TSBYE	HP	1.500000%	20.0	22.0	3.80	0.20	3
Unclear	Planktonic	TSBYE	HP	1.500000%	20.0	22.0	3.80	0.20	3
Unclear	Planktonic	TSBYE	BAC	0.005000%	20.0	22.0	3.60	0.30	3
Unclear	Planktonic	TSBYE	BAC	0.005000%	20.0	22.0	5.20	0.30	3
Unclear	Planktonic	TSBYE	BAC	0.005000%	20.0	22.0	5.90	0.30	3
Unclear	Planktonic	TSBYE	HP	1.500000%	20.0	22.0	4.70	0.30	3
Unclear	Planktonic	TSBYE	BAC	0.005000%	20.0	22.0	4.20	0.30	3
Unclear	Planktonic	TSBYE	HP	1.500000%	20.0	22.0	3.70	0.30	3
Unclear	Planktonic	TSBYE	BAC	0.005000%	20.0	22.0	5.20	0.30	3
Unclear	Planktonic	TSBYE	BAC	0.005000%	20.0	22.0	4.70	0.30	3
Unclear	Planktonic	TSBYE	BAC	0.005000%	20.0	22.0	3.20	0.30	3
Unclear	Planktonic	TSBYE	HP	1.500000%	20.0	22.0	5.20	0.40	3
Unclear	Planktonic	TSBYE	BAC	0.005000%	20.0	22.0	3.80	0.40	3
Unclear	Planktonic	TSBYE	HP	1.500000%	20.0	22.0	1.50	0.40	3
Unclear	Planktonic	TSBYE	BAC	0.005000%	20.0	22.0	5.60	0.40	3
Unclear	Planktonic	TSBYE	HP	1.500000%	20.0	22.0	3.40	0.40	3
Unclear	Planktonic	TSBYE	HP	1.500000%	20.0	22.0	0.60	0.40	3

Unclear	Planktonic	TSBYE	HP	1.500000%	20.0	22.0	3.40	0.40	3	
Unclear	Planktonic	TSBYE	BAC	0.005000%	20.0	22.0	3.30	0.40	3	
Unclear	Planktonic	TSBYE	HP	1.500000%	20.0	22.0	3.20	0.40	3	
Unclear	Planktonic	TSBYE	HP	1.500000%	20.0	22.0	2.50	0.50	3	
Unclear	Planktonic	TSBYE	HP	1.500000%	20.0	22.0	3.30	0.60	3	
Unclear	Planktonic	TSBYE	BAC	0.005000%	20.0	22.0	3.20	0.60	3	
Unclear	Planktonic	TSBYE	BAC	0.000100%	20.0	22.0	4.70	0.60	3	
Unclear	Planktonic	TSBYE	HP	1.500000%	20.0	22.0	2.20	0.60	3	
Unclear	Planktonic	TSBYE	BAC	0.005000%	20.0	22.0	4.20	0.60	3	
Unclear	Planktonic	TSBYE	HP	1.500000%	20.0	22.0	2.80	0.60	3	
Unclear	Planktonic	TSBYE	BAC	0.005000%	20.0	22.0	3.90	0.60	3	
Unclear	Planktonic	TSBYE	BAC	0.005000%	20.0	22.0	3.90	0.60	3	
Unclear	Planktonic	TSBYE	BAC	0.005000%	20.0	22.0	4.10	0.70	3	
Unclear	Planktonic	TSBYE	BAC	0.005000%	20.0	22.0	5.50	0.80	3	
Unclear	Planktonic	TSBYE	BAC	0.005000%	20.0	22.0	4.60	0.80	3	
Unclear	Planktonic	TSBYE	HP	1.500000%	20.0	22.0	2.40	0.80	3	
Unclear	Planktonic	TSBYE	BAC	0.005000%	20.0	22.0	4.30	0.80	3	
Unclear	Planktonic	TSBYE	BAC	0.005000%	20.0	22.0	2.80	0.90	3	
Unclear	Planktonic	TSBYE	HP	1.500000%	20.0	22.0	3.60	0.90	3	
Unclear	Planktonic	TSBYE	BAC	0.005000%	20.0	22.0	4.20	0.90	3	
Unclear	Planktonic	TSBYE	BAC	0.005000%	20.0	22.0	3.60	0.90	3	
Unclear	Planktonic	TSBYE	BAC	0.005000%	20.0	22.0	3.40	0.90	3	
Unclear	Planktonic	TSBYE	BAC	0.005000%	20.0	22.0	3.30	1.00	3	
Unclear	Planktonic	TSBYE	HP	1.500000%	20.0	22.0	3.50	1.10	3	
Unclear	Planktonic	TSBYE	HP	1.500000%	20.0	22.0	4.00	1.10	3	
Unclear	Planktonic	TSBYE	HP	1.500000%	20.0	22.0	3.80	1.10	3	
Unclear	Planktonic	TSBYE	HP	1.500000%	20.0	22.0	3.00	1.20	3	
Unclear	Planktonic	TSBYE	BAC	0.005000%	20.0	22.0	3.40	1.30	3	
Unclear	Planktonic	TSBYE	BAC	0.005000%	20.0	22.0	3.60	1.30	3	
Unclear	Planktonic	TSBYE	HP	1.500000%	20.0	22.0	3.60	1.50	3	
Unclear	Planktonic	TSBYE	HP	1.500000%	20.0	22.0	3.40	1.70	3	
V7	Planktonic	PBS	SH	0.010000%	3.0	4.0	5.58	0.04	3	[43]

Scott A	Planktonic	PBS	SH	0.010000%	0.5	25.0	4.68	0.11	3	
Scott A	Planktonic	PBS	SH	0.010000%	0.5	4.0	3.85	0.18	3	
Scott A	Planktonic	PBS	SH	0.010000%	3.0	4.0	5.55	0.18	3	
Scott A	Planktonic	PBS	SH	0.010000%	3.0	25.0	5.33	0.21	3	
V7	Planktonic	PBS	SH	0.010000%	0.5	25.0	6.11	0.64	3	
V7	Planktonic	PBS	SH	0.010000%	0.5	4.0	4.34	0.91	3	
ATCC 19115	Planktonic	Sterile water	PAA	1.000000%	5.0	25.0	4.30	0.00	3	[17]
ATCC 19115	Planktonic	Sterile water	PAA	1.000000%	10.0	25.0	8.80	0.00	3	
ATCC 19115	Planktonic	Sterile water	PAA	1.000000%	15.0	25.0	8.80	0.00	3	
ATCC 19115	Planktonic	Sterile water	PAA	0.500000%	5.0	50.0	4.20	0.00	3	
ATCC 19115	Planktonic	Sterile water	PAA	0.500000%	10.0	50.0	8.70	0.00	3	
ATCC 19115	Planktonic	Sterile water	PAA	0.500000%	15.0	50.0	8.70	0.00	3	
ATCC 19115	Planktonic	Sterile water	PAA	0.300000%	5.0	70.0	5.20	0.00	3	
ATCC 19115	Planktonic	Sterile water	PAA	0.300000%	15.0	70.0	8.80	0.00	3	
ATCC 19115	Planktonic	Sterile water	PAA	0.300000%	10.0	70.0	6.35	0.07	3	
Cocktail	Planktonic	BPW	EW	0.005556%	2.0	22.0	7.84	0.27	3	[114]
Cocktail	Planktonic	BPW	EW	0.007000%	2.0	22.0	7.70	0.61	3	
Cocktail	Planktonic	BPW	EW	0.004442%	2.0	22.0	7.62	0.65	3	
Cocktail	Planktonic	BPW	EW	0.006330%	2.0	22.0	7.48	0.79	3	
10403S	Planktonic	PBS	CDS	0.030000%	12.5	37.0	0.17	0.05	3	[115]
10403S	Planktonic	PBS	CDS	0.030000%	10.0	37.0	0.06	0.10	3	
10403S	Planktonic	PBS	CDS	0.010000%	15.0	37.0	0.25	0.11	3	
10403S	Planktonic	PBS	CDS	0.010000%	12.5	37.0	0.34	0.13	3	
10403S	Planktonic	PBS	CDS	0.030000%	20.0	37.0	0.21	0.15	3	
10403S	Planktonic	PBS	CDS	0.010000%	10.0	37.0	0.34	0.16	3	
10403S	Planktonic	PBS	CDS	0.030000%	7.5	37.0	0.06	0.17	3	
10403S	Planktonic	PBS	CDS	0.010000%	20.0	37.0	0.20	0.18	3	
10403S	Planktonic	PBS	CDS	0.010000%	5.0	37.0	0.31	0.19	3	
10403S	Planktonic	PBS	CDS	0.010000%	7.5	37.0	0.34	0.19	3	
10403S	Planktonic	PBS	CDS	0.010000%	2.5	37.0	0.14	0.30	3	
10403S	Planktonic	PBS	CDS	0.030000%	15.0	37.0	0.33	0.37	3	
10403S	Planktonic	PBS	CDS	0.030000%	5.0	37.0	0.13	0.38	3	

10403S	Planktonic	PBS	CDS	0.050000%	15.0	37.0	3.65	0.55	3	
10403S	Planktonic	PBS	CDS	0.050000%	20.0	37.0	3.65	0.55	3	
10403S	Planktonic	PBS	CDS	0.050000%	2.5	37.0	0.90	0.62	3	
10403S	Planktonic	PBS	CDS	0.050000%	12.5	37.0	2.59	1.07	3	
10403S	Planktonic	PBS	CDS	0.050000%	10.0	37.0	1.97	1.42	3	
10403S	Planktonic	PBS	CDS	0.050000%	7.5	37.0	1.65	1.45	3	
10403S	Planktonic	PBS	CDS	0.050000%	5.0	37.0	1.45	1.51	3	
Cocktail	Planktonic	PBS	SHD	0.600000%	1.0	22.5	0.26	0.03	3	[116]
Cocktail	Planktonic	PBS	SHD	0.400000%	1.0	22.5	0.19	0.05	3	
Cocktail	Planktonic	PBS	SHD	0.400000%	2.0	22.5	0.27	0.05	3	
Cocktail	Planktonic	PBS	SHD	0.600000%	2.0	22.5	0.33	0.05	3	
Cocktail	Planktonic	PBS	SHD	0.800000%	1.0	22.5	0.30	0.07	3	
Cocktail	Planktonic	PBS	SHD	1.000000%	1.0	22.5	0.51	0.11	3	
Cocktail	Planktonic	PBS	SHD	0.800000%	2.0	22.5	0.43	0.18	3	
Cocktail	Planktonic	PBS	SHD	1.000000%	2.0	22.5	1.25	0.20	3	
Cocktail	Planktonic	PBS	SHD	1.400000%	1.0	22.5	2.71	0.26	3	
Cocktail	Planktonic	PBS	SHD	1.400000%	2.0	22.5	5.44	0.27	3	
Cocktail	Planktonic	PBS	SHD	1.200000%	1.0	22.5	0.97	0.33	3	
Cocktail	Planktonic	PBS	SHD	1.200000%	2.0	22.5	3.31	0.50	3	
ATCC 19115	Planktonic	Deionized water	EW	0.000500%	1.0	23.0	5.20	Unclear	3	[75]
ATCC 19115	Planktonic	Deionized water	EW	0.000500%	3.0	23.0	5.18	Unclear	3	
ATCC 19115	Planktonic	Deionized water	EW	0.000500%	5.0	23.0	4.91	Unclear	3	
ATCC 19115	Planktonic	Deionized water	EW	0.000500%	7.0	23.0	4.62	Unclear	3	
ATCC 19115	Planktonic	Deionized water	EW	0.000500%	10.0	23.0	4.23	Unclear	3	
ATCC 19115	Planktonic	Deionized water	EW	0.005000%	1.0	23.0	4.92	Unclear	3	
ATCC 19115	Planktonic	Deionized water	EW	0.000680%	1.0	23.0	5.40	Unclear	3	
ATCC 19115	Planktonic	Deionized water	EW	0.000620%	1.0	23.0	5.30	Unclear	3	
ATCC 19115	Planktonic	Deionized water	EW	0.000560%	1.0	23.0	5.20	Unclear	3	
ATCC 19115	Planktonic	Deionized water	EW	0.000500%	1.0	23.0	5.30	Unclear	3	
ATCC 19115	Planktonic	Deionized water	EW	0.000320%	1.0	23.0	2.23	Unclear	3	
ATCC 19115	Planktonic	Deionized water	EW	0.005000%	1.0	23.0	4.90	Unclear	3	
ATCC 19115	Planktonic	Deionized water	EW	0.000480%	1.0	4.0	4.98	Unclear	3	

ATCC 19115	Planktonic	Deionized water	EW	0.000490%	1.0	15.0	5.00	Unclear	3	
ATCC 19115	Planktonic	Deionized water	EW	0.000500%	1.0	23.0	5.20	Unclear	3	
ATCC 19115	Planktonic	Deionized water	EW	0.000520%	1.0	35.0	6.20	Unclear	3	
ATCC 19115	Planktonic	Deionized water	EW	0.000550%	1.0	50.0	7.42	Unclear	3	
ATCC 19115	Planktonic	Deionized water	EW	0.005100%	1.0	35.0	6.00	Unclear	3	
ATCC 19115	Planktonic	Saline solution	EW	0.000500%	1.0	22.5	5.20	0.04	3	[117]
ATCC 19115	Planktonic	Saline solution	EW	0.001000%	1.0	22.5	6.28	0.05	3	
ATCC 19115	Planktonic	Saline solution	EW	0.000700%	0.5	22.5	5.61	0.07	3	
ATCC 19115	Planktonic	Saline solution	EW	0.005000%	1.5	22.5	6.25	0.07	3	
ATCC 19115	Planktonic	Saline solution	EW	0.000500%	1.0	22.5	5.60	0.08	3	
ATCC 19115	Planktonic	Saline solution	EW	0.000500%	1.5	22.5	6.14	0.08	3	
ATCC 19115	Planktonic	Saline solution	EW	0.005000%	0.5	22.5	5.41	0.08	3	
ATCC 19115	Planktonic	Saline solution	EW	0.001000%	1.5	22.5	6.79	0.09	3	
ATCC 19115	Planktonic	Saline solution	EW	0.005000%	1.0	22.5	5.72	0.09	3	
ATCC 19115	Planktonic	Saline solution	EW	0.001000%	1.0	22.5	5.58	0.10	3	
ATCC 19115	Planktonic	Saline solution	EW	0.001000%	0.5	22.5	5.94	0.10	3	
ATCC 19115	Planktonic	Saline solution	EW	0.000700%	1.5	22.5	6.44	0.11	3	
ATCC 19115	Planktonic	Saline solution	EW	0.000700%	1.0	22.5	5.90	0.12	3	
ATCC 19115	Planktonic	Saline solution	EW	0.000500%	0.5	22.5	5.32	0.18	3	
Scott A	Planktonic	PBS	SH	0.051200%	5.0	22.0	8.00	Unclear	3	[79]
Scott A	Planktonic	PBS	SH	0.025600%	5.0	22.0	8.00	Unclear	3	
Scott A	Planktonic	PBS	SH	0.012800%	5.0	22.0	8.00	Unclear	3	
Scott A	Planktonic	PBS	CHD	0.025600%	5.0	22.0	8.00	Unclear	3	
Scott A	Planktonic	PBS	CHD	0.012800%	5.0	22.0	8.00	Unclear	3	
Scott A	Planktonic	PBS	BAC	0.025600%	5.0	22.0	8.00	Unclear	3	
Scott A	Planktonic	PBS	BAC	0.012800%	5.0	22.0	8.00	Unclear	3	
ATCC 7644	Planktonic	Saline solution	EW	0.011000%	0.5	22.5	8.67	0.00	1	[118]
ATCC 19111	Planktonic	Hard water	BAAC	0.010000%	5.0	22.5	7.23	Unclear	3	[119]
Vegetable isolated	Planktonic	Hard water	BAAC	0.010000%	5.0	22.5	6.96	Unclear	3	
Meat isolated	Planktonic	Hard water	BAAC	0.004000%	5.0	22.5	6.84	Unclear	3	
Dairy isolated	Planktonic	Hard water	BAAC	0.004000%	5.0	22.5	6.92	Unclear	3	

Fish isolated	Planktonic	Hard water	BAAC	0.004000%	5.0	22.5	6.76	Unclear	3	
Clinical isolated	Planktonic	Hard water	BAAC	0.010000%	5.0	22.5	7.07	Unclear	3	
ATCC 19111	Planktonic	Hard water	BAAC	12.800000%	5.0	22.5	7.05	Unclear	3	
Vegetable isolated	Planktonic	Hard water	BAAC	12.800000%	5.0	22.5	6.78	Unclear	3	
Meat isolated	Planktonic	Hard water	BAAC	12.800000%	5.0	22.5	6.62	Unclear	3	
Dairy isolated	Planktonic	Hard water	BAAC	12.800000%	5.0	22.5	6.75	Unclear	3	
Fish isolated	Planktonic	Hard water	BAAC	12.800000%	5.0	22.5	6.50	Unclear	3	
Clinical isolated	Planktonic	Hard water	BAAC	12.800000%	5.0	22.5	6.90	Unclear	3	
ATCC 19111	Planktonic	Hard water	CDS	0.000050%	5.0	22.5	7.67	Unclear	3	
Vegetable isolated	Planktonic	Hard water	CDS	0.000050%	5.0	22.5	7.39	Unclear	3	
Meat isolated	Planktonic	Hard water	CDS	0.000050%	5.0	22.5	7.23	Unclear	3	
Dairy isolated	Planktonic	Hard water	CDS	0.000050%	5.0	22.5	7.33	Unclear	3	
Fish isolated	Planktonic	Hard water	CDS	0.000050%	5.0	22.5	7.06	Unclear	3	
Clinical isolated	Planktonic	Hard water	CDS	0.000050%	5.0	22.5	7.52	Unclear	3	
ATCC 19111	Planktonic	Hard water	CH	24.000000%	5.0	22.5	7.31	Unclear	3	
Vegetable isolated	Planktonic	Hard water	CH	32.000000%	5.0	22.5	6.95	Unclear	3	
Meat isolated	Planktonic	Hard water	CH	36.000000%	5.0	22.5	6.71	Unclear	3	
Dairy isolated	Planktonic	Hard water	CH	32.000000%	5.0	22.5	6.93	Unclear	3	
Fish isolated	Planktonic	Hard water	CH	28.000000%	5.0	22.5	6.58	Unclear	3	
Clinical isolated	Planktonic	Hard water	CH	40.000000%	5.0	22.5	7.08	Unclear	3	
Cocktail	Planktonic	BPW	EW	0.002500%	0.3	22.5	1.67	Unclear	3	[120]
Cocktail	Planktonic	BPW	EW	0.005000%	0.3	22.5	3.72	Unclear	3	
Cocktail	Planktonic	BPW	EW	0.010000%	0.3	22.5	7.36	Unclear	3	
Cocktail	Planktonic	Deionized water	EW	0.007330%	2.0	35.0	7.91	0.00	3	[121]
Cocktail	Planktonic	Deionized water	EW	0.007330%	1.0	45.0	7.91	0.00	3	
Cocktail	Planktonic	Deionized water	EW	0.004850%	5.0	23.0	6.64	0.33	3	
Cocktail	Planktonic	Deionized water	EW	0.004300%	5.0	4.0	6.57	0.37	3	
Cocktail	Planktonic	Deionized water	CA	5.000000%	1.0	25.0	0.66	0.06	6	[122]
Cocktail	Planktonic	Deionized water	AA	2.500000%	1.0	25.0	0.75	0.10	6	

Cocktail	Planktonic	Deionized water	AA	2.500000%	10.0	25.0	0.76	0.10	6
Cocktail	Planktonic	Deionized water	HP	1.500000%	10.0	25.0	3.58	0.11	6
Cocktail	Planktonic	Deionized water	CA	5.000000%	10.0	25.0	1.83	0.17	6
Cocktail	Planktonic	Deionized water	HP	1.500000%	1.0	25.0	1.75	0.23	6
Cocktail	Planktonic	Deionized water	AA	5.000000%	1.0	25.0	1.80	0.29	6
Cocktail	Planktonic	Deionized water	AA	5.000000%	10.0	25.0	5.57	0.32	6
Cocktail	Planktonic	Deionized water	HP	3.000000%	10.0	25.0	5.42	0.37	6
Cocktail	Planktonic	Deionized water	AA	2.500000%	10.0	55.0	5.06	0.45	6
Cocktail	Planktonic	Deionized water	HP	3.000000%	1.0	25.0	3.77	0.55	6
Cocktail	Planktonic	Deionized water	AA	2.500000%	1.0	55.0	2.97	1.29	6
